# Supplementary material for: Scutellaria Barbata inhibits epithelial-mesenchymal transformation through PI3K/AKT and MDM2 thus inhibiting the proliferation, migration and promoting apoptosis of Cervical Cancer cells
Source: PLoS One. 2025 Apr 16;20(4):e0321556. doi: 10.1371/journal.pone.0321556 (PMC12002470; doi:10.1371/journal.pone.0321556)
Supplement: S1 Table — (PDF) [file pone.0321556.s001.pdf]

Table1. Sequences of primers used for real-time RT-PCR

Name	Forward (5'-3')	Reverse (5'-3')	note
GADPH	TGC CAT GTA GAC CCC TTG AAG	ATG GTA CAT GAC AAG GTG CGG	10.1038/nm.2863
TNF-α	CTC TTC TGC CTG CTG CAC TTT G	ATG GGC TAC AGG CTT GTC ACT C	10.1016/j.celrep.2017.08.003
PI3K	GGG GAT GAT TTA CGG CAA GAT A	CAC CAC CTC AAT AAG TCC CAC A	PMID: 31934074
AKT	GCA GCA CGT GTA CGA GAA GA	GGT GTC AGT CTC CGA CGT G	PMID: 31934074
VEGFA	TTG CTG CTC TAC CTC CAC CAT	GGT GAT GTT GGA CTC CTC AGT G	10.1002/kjm2.12127
EGFR	AAC ACC CTG GTC TGG AAG TAC G	TCG TTG GAC AGC CTT CAA GAC C	10.1007/s11010-019-03575-y
MDM2	AGG AGA TTT GTT TGG CGT GC	TGA GTC CGA TGA TTC CTG CTG	10.1038/nm.2863
