# Supplementary material for: Scutellaria Barbata inhibits epithelial-mesenchymal transformation through PI3K/AKT and MDM2 thus inhibiting the proliferation, migration and promoting apoptosis of Cervical Cancer cells
Source: PLoS One. 2025 Apr 16;20(4):e0321556. doi: 10.1371/journal.pone.0321556 (PMC12002470; doi:10.1371/journal.pone.0321556)
Supplement: Attachment — Submitted filename: PONE-D-24-33635.pdf [file pone.0321556.s002.pdf]

PLOS ONE

Scutellaria Barbata inhibits epithelial-mesenchymal transformation through PI3K/AKT and MDM2 thus inhibiting the proliferation, migration and promoting apoptosis of Cervical Cancer cells

--Manuscript Draft--

**Manuscript Number:**	PONE-D-24-33635
**Article Type:**	Research Article
**Full Title:**	Scutellaria Barbata inhibits epithelial-mesenchymal transformation through PI3K/AKT and MDM2 thus inhibiting the proliferation, migration and promoting apoptosis of Cervical Cancer cells
**Short Title:**	Scutellaria Barbata inhibits epithelial-mesenchymal transformation on Cervical Cancer cells
**Corresponding Author:**	wen-qi feng<br>Yibin Hospital of T.C.M<br>Yibin, sichuan CHINA
**Keywords:**	Scutellaria Barbata; cervical cancer; PI3K/AKT signaling pathway; bioinformatics
**Abstract:**	Background: Cervical cancer is the fourth most common malignant tumor among women with high morbidity and mortality. We found the active ingredients, action targets and pathways of Scutellaria Barbata against cervical cancer cells, and verified its effects and mechanisms on the proliferation, migration and apoptosis of cervical cancer cells through cellular experiments.<br>Materials and methods: The potential effective components of Scutellaria Barbata were obtained by data mining. CCK 8 experiments were used to verify the optimal action time and concentration of the key active components, Baicalin and Wogonin on Hela cells. Transwell Experiments to verify the migration rate of Hela cells at different time points of action. Hela cell apoptosis was determined by a Tunel assay. RT-qPCR and Western Blot detection of related genes and protein expression after the effects of different concentrations of Baicalein and Wogonin on Hela cells.<br>Results: Obtained 27 core intersecting targets. Four genes associated with survival of cervical cancer patients were selected by survival by univariate analysis: EGFR, TNF-α, VEGFA and MDM2 (P<0.1). GO and KEGG pathway enrichment analysis showed the four genes played positive regulatory roles, mainly enriched in cellular extracellular zone and cytoplasm. Pathways include cancer pathways, proteoglycans and PI3K/AKT signal. CCK 8 experiments showed that the proliferation rate of Hela cells was significantly lower than that of the control group, and the optimal drug concentration was 10 mg/mL (P <0.001). The migration assay showed that the incubation of the medium with a mixture of 10 mg/mL each or 72h of 5 ml/mL each significantly inhibited the migration of Hela cells (P <0.001). Tunel results showed that single-agent Baicalein or Wogonin 10 mg/mL had the highest rate of apoptosis on Hela cells after culturing the cells for 72 h. The results showed the highest rate of apoptosis on Hela cells. Baicalein or Wogonin (10 mg/mL + 10 mg/mL) had the highest apoptosis ratio in Hela cells after 72h and a higher combination ratio than in the single agent group (P <0.001). The difference between the single drug group and the corresponding low dose group was significant (P<0.001). After Hela cells were cultured for 72 h, the mRNA and protein expressions corresponding to EGFR, TNF-α, VEGFA, PI3K, AKT and MDM2 were decreased in the experimental group compared with the control group as confirmed by RT-PCR and Western blot, and the extent of the decrease was more obvious with the increase of the drug concentration, and under the condition of the same drug dosage, the effect of the Baicalein group was more pronounced than that of the Wogonin group, and the effect was more obvious in the two-drug group compared with the single-drug group (P<0.05).<br>Conclusion: After combining with EGFR, TNF-α and VEGFA, the active ingredient of Sculellaria Barbata can regulate the PI3K/AKT signaling pathway and down-regulate the expression of MDM2 gene to inhibit the epithelial-mesenchymal transition, which can inhibit cervical cancer cell proliferation, migration, and promote their apoptosis. Sculellaria Barbata is a potential therapeutic candidate for cervical cancer.
**Order of Authors:**	wen-qi feng

Powered by Editorial Manager® and ProduXion Manager® from Aries Systems Corporation

	Guobing Wang
	Congchao Jia
	Dexin Wang
	Lan Li
	Gang Tian

Additional Information:	
**Question**	**Response**
**Financial Disclosure**<br><br>Enter a financial disclosure statement that describes the sources of funding for the work included in this submission. Review the submission guidelines for detailed requirements. View published research articles from *PLOS ONE* for specific examples.<br><br>This statement is required for submission and **will appear in the published article** if the submission is accepted. Please make sure it is accurate.<br><br>**Funded studies**<br>Enter a statement with the following details:<br>• Initials of the authors who received each award<br>• Grant numbers awarded to each author<br>• The full name of each funder<br>• URL of each funder website<br>• Did the sponsors or funders play any role in the study design, data collection and analysis, decision to publish, or preparation of the manuscript?<br><br>Did you receive funding for this work?	Yes
Please add funding details.<br>as follow-up to "**Financial Disclosure**<br><br>Enter a financial disclosure statement that describes the sources of funding for the work included in this submission. Review the submission guidelines for detailed requirements. View published research articles from *PLOS ONE* for specific examples.	This study was supported by grants from the Luzhou Science and Technology Department Applied Basic Research Program (No: 2022-JYJ-145), the Sichuan Province Science and Technology Department of foreign (border) high-end talent introduction project (No: 2023JDGD0037), Sichuan Provincial Medical Association (No: Q22027), YiBin Science and Technology Department Social Development Projects (No:2022SF004), and Medical Scientific Research Project of Yibin Municipal Health Commission (No:2023YW026).

This statement is required for submission and **will appear in the published article** if the submission is accepted. Please make sure it is accurate.<br><br>**Funded studies**<br>Enter a statement with the following details:<br>• Initials of the authors who received each award<br>• Grant numbers awarded to each author<br>• The full name of each funder<br>• URL of each funder website<br>• Did the sponsors or funders play any role in the study design, data collection and analysis, decision to publish, or preparation of the manuscript?<br><br>Did you receive funding for this work?"	
Please select the country of your main research funder (please select carefully as in some cases this is used in fee calculation).<br>as follow-up to "**Financial Disclosure**<br><br>Enter a financial disclosure statement that describes the sources of funding for the work included in this submission. Review the submission guidelines for detailed requirements. View published research articles from *PLOS ONE* for specific examples.<br><br>This statement is required for submission and **will appear in the published article** if the submission is accepted. Please make sure it is accurate.	CHINA - CN

**Funded studies**<br>Enter a statement with the following details:<br>• Initials of the authors who received each award<br>• Grant numbers awarded to each author<br>• The full name of each funder<br>• URL of each funder website<br>• Did the sponsors or funders play any role in the study design, data collection and analysis, decision to publish, or preparation of the manuscript?<br><br>Did you receive funding for this work?"	
**Competing Interests**<br><br>Use the instructions below to enter a competing interest statement for this submission. On behalf of all authors, disclose any competing interests that could be perceived to bias this work—acknowledging all financial support and any other relevant financial or non-financial competing interests.<br><br>This statement is required for submission and **will appear in the published article** if the submission is accepted. Please make sure it is accurate and that any funding sources listed in your Funding Information later in the submission form are also declared in your Financial Disclosure statement.<br><br>View published research articles from *PLOS ONE* for specific examples.	The authors declare that the research was conducted in the absence of any commercial or financial relationships that could be construed as a potential conflict of interest.

Powered by Editorial Manager® and ProduXion Manager® from Aries Systems Corporation

**NO authors have competing interests**<br><br>Enter: *The authors have declared that no competing interests exist.*<br><br>**Authors with competing interests**<br><br>Enter competing interest details beginning with this statement:<br><br>*I have read the journal's policy and the authors of this manuscript have the following competing interests: [insert competing interests here]*<br><br>* typeset	
**Ethics Statement**<br><br>Enter an ethics statement for this submission. This statement is required if the study involved:<br><br>• Human participants<br>• Human specimens or tissue<br>• Vertebrate animals or cephalopods<br>• Vertebrate embryos or tissues<br>• Field research<br><br>Write "N/A" if the submission does not require an ethics statement.<br><br>General guidance is provided below. Consult the submission guidelines for detailed instructions. **Make sure that all information entered here is included in the Methods section of the manuscript.**	N/A

Powered by Editorial Manager® and ProduXion Manager® from Aries Systems Corporation

**Format for specific study types**<br><br>**Human Subject Research (involving human participants and/or tissue)**<br>• Give the name of the institutional review board or ethics committee that approved the study<br>• Include the approval number and/or a statement indicating approval of this research<br>• Indicate the form of consent obtained (written/oral) or the reason that consent was not obtained (e.g. the data were analyzed anonymously)<br><br>**Animal Research (involving vertebrate animals, embryos or tissues)**<br>• Provide the name of the Institutional Animal Care and Use Committee (IACUC) or other relevant ethics board that reviewed the study protocol, and indicate whether they approved this research or granted a formal waiver of ethical approval<br>• Include an approval number if one was obtained<br>• If the study involved *non-human primates*, add *additional details* about animal welfare and steps taken to ameliorate suffering<br>• If anesthesia, euthanasia, or any kind of animal sacrifice is part of the study, include briefly which substances and/or methods were applied<br><br>**Field Research**<br><br>Include the following details if this study involves the collection of plant, animal, or other materials from a natural setting:<br>• Field permit number<br>• Name of the institution or relevant body that granted permission	
**Data Availability**<br><br>Authors are required to make all data underlying the findings described fully available, without restriction, and from the time of publication. PLOS allows rare exceptions to address legal and ethical concerns. See the PLOS Data Policy and FAQ for detailed information.	Yes - all data are fully available without restriction

Powered by Editorial Manager® and ProduXion Manager® from Aries Systems Corporation

A Data Availability Statement describing where the data can be found is required at submission. Your answers to this question constitute the Data Availability Statement and **will be published in the article**, if accepted.<br><br>**Important:** Stating 'data available on request from the author' is not sufficient. If your data are only available upon request, select 'No' for the first question and explain your exceptional situation in the text box.<br><br>Do the authors confirm that all data underlying the findings described in their manuscript are fully available without restriction?	
**Describe where the data may be found in full sentences. If you are copying our sample text, replace any instances of XXX with the appropriate details.**<br><br>• If the data are **held or will be held in a public repository**, include URLs, accession numbers or DOIs. If this information will only be available after acceptance, indicate this by ticking the box below. For example: *All XXX files are available from the XXX database (accession number(s) XXX, XXX.*).<br>• If the data are all contained **within the manuscript and/or Supporting Information files**, enter the following: *All relevant data are within the manuscript and its Supporting Information files.*<br>• If neither of these applies but you are able to provide **details of access elsewhere**, with or without limitations, please do so. For example:<br><br>*Data cannot be shared publicly because of [XXX]. Data are available from the XXX Institutional Data Access / Ethics Committee (contact via XXX) for researchers who meet the criteria for access to confidential data.*<br><br>*The data underlying the results presented in the study are available from (include the name of the third party*	All relevant data are within the manuscript and its Supporting Information files.

and contact information or URL).<br>• This text is appropriate if the data are owned by a third party and authors do not have permission to share the data.<br><br>* typeset	
Additional data availability information:	

Manuscript
Click here to access/download;Manuscript;manuscript.pdf

Scutellaria Barbata inhibits epithelial-mesenchymal transformation through PI3K/AKT and MDM2 thus inhibiting the proliferation, migration and promoting apoptosis of Cervical Cancer cells

Guobing Wang[1¶], Congchao Jia[2¶], Dexin Wang[2,3,4], Lan Li[2,3,4], Gang Tian[2,3,4,*] and Wenqi Feng[1,*]

[1] Medical Research Laboratory, YiBin Hospital of T.C.M, Yibin, China.

[2] Department of Laboratory Medicine, The Affiliated Hospital of Southwest Medical University, Luzhou, China.

[3] Sichuan Province Engineering Technology Research Center of Molecular Diagnosis of Clinical Diseases, Luzhou, China.

[4] Molecular Diagnosis of Clinical Diseases Key Laboratory of Luzhou, Luzhou, China.

* Corresponding author

E-Mail: tiangang@swmu.edu.cn(Gang Tian) and E-Mail: moonly1981@163.com(Wenqi Feng)

¶These authors contributed equally to this work and share first authorship.

ABSTRACT

Background: Cervical cancer is the fourth most common malignant tumor among women with high morbidity and mortality. We found the active ingredients, action targets and pathways of Scutellaria Barbata against cervical cancer cells, and verified its effects and mechanisms on the proliferation, migration and apoptosis of cervical cancer cells through cellular experiments.

Materials and methods: The potential effective components of Scutellaria Barbata were obtained by data mining. CCK 8 experiments were used to verify the optimal action time and concentration of the key active components, Baicalin and Wogonin on Hela cells. Transwell Experiments to verify the

migration rate of Hela cells at different time points of action. Hela cell apoptosis was determined by a Tunel assay. RT-qPCR and Western Blot detection of related genes and protein expression after the effects of different concentrations of Baicalein and Wogonin on Hela cells.

Results: Obtained 27 core intersecting targets. Four genes associated with survival of cervical cancer patients were selected by survival by univariate analysis: EGFR, TNF-α, VEGFA and MDM2 ($P<0.1$). GO and KEGG pathway enrichment analysis showed the four genes played positive regulatory roles, mainly enriched in cellular extracellular zone and cytoplasm. Pathways include cancer pathways, proteoglycans and PI3K/AKT signal. CCK 8 experiments showed that the proliferation rate of Hela cells was significantly lower than that of the control group, and the optimal drug concentration was 10 mg/mL ($P <0.001$). The migration assay showed that the incubation of the medium with a mixture of 10 mg/mL each or 72h of 5 ml/mL each significantly inhibited the migration of Hela cells ($P <0.001$). Tunel results showed that single-agent Baicalein or Wogonin 10 mg/mL had the highest rate of apoptosis on Hela cells after culturing the cells for 72 h. The results showed the highest rate of apoptosis on Hela cells. Baicalein or Wogonin (10 mg/mL + 10 mg/mL) had the highest apoptosis ratio in Hela cells after 72h and a higher combination ratio than in the single agent group ($P <0.001$). The difference between the single drug group and the corresponding low dose group was significant ($P<0.001$). After Hela cells were cultured for 72 h, the mRNA and protein expressions corresponding to EGFR, TNF-α, VEGFA, PI3K, AKT and MDM2 were decreased in the experimental group compared with the control group as confirmed by RT-PCR and Western blot, and the extent of the decrease was more obvious with the increase of the drug concentration, and under the condition of the same drug dosage, the effect of the Baicalein group was more pronounced than that of the Wogonin group, and the effect was more obvious in the two-drug

group compared with the single-drug group (P<0.05).

Conclusion: After combining with EGFR, TNF-α and VEGFA, the active ingredient of Sculellaria Barbata can regulate the PI3K/AKT signaling pathway and down-regulate the expression of MDM2 gene to inhibit the epithelial-mesenchymal transition, which can inhibit cervical cancer cell proliferation, migration, and promote their apoptosis. Sculellaria Barbata is a potential therapeutic candidate for cervical cancer.

Keywords: Scutellaria Barbata; cervical cancer; PI3K/AKT signaling pathway; bioinformatics

1. Introduction

As a common malignant tumor among women, cervical cancer poses a serious risk to women's physical and mental wellbeing because of its high malignancy, incidence, and recurrence rate after surgery(1). In 2018, there were about 570,000 new cases of i worldwide, accounting for 3.15% of the incidence of all malignant tumors, and about 310,000 deaths, accounting for 3.26% of the total number of deaths from all malignant tumors. The incidence rate in China is about 3 ‰, and the incidence rate accounts for 18.6% of the global malignant tumors. It has been shown that persistent high-risk HPV infection is closely associated with cervical cancer and precancerous lesions, which effectively reduces the clinical underdiagnosis and misdiagnosis rate and improves the cervical cancer accuracy(2). However, combined screening also has certain false-negative and false-positive rates, and the peak age of cervical cancer patients is showing a younger trend(3). Therefore, preventing and treating cervical cancer remains a clinical challenge.

Currently, surgery, radiotherapy, and chemotherapy are the main treatments for cervical cancer, and Chinese medicine can also be used as adjuvant treatment(4). The advantages of surgical treatment are

that the cancer can be completely removed in one operation in early stage cases, the treatment time is short, and the ovarian function of normal patients can be preserved(5). However, some patients may have serious complications during surgical treatment, slow postoperative recovery, and even sequelae(6). Radiotherapy is applicable to all patients with cervical cancer, and while killing cancer cells, radiotherapy can also damage normal tissues in the irradiated field, with complications in the rectum and bladder being the most common(7). Chemotherapy is a treatment method targeting the systemic level and is mainly used for advanced cases of cervical cancer or combined surgical treatment and radiation therapy, and can also be used to treat recurrent tumors(8). However, chemotherapy has certain toxic side effects, including nausea, vomiting, loss of appetite and gastrointestinal and bone marrow suppressive reactions such as decreased white blood cells and platelets(9). In contrast, TCM with surgery, radiation and chemotherapy for cervical cancer has obvious advantages, not only in reducing the side effects caused by radiation therapy or chemotherapy, but also in improving the efficacy and accelerating the recovery of patients, so it is worth further exploring its mechanism of action(10).

There have been a growing number of studies showing the anti-tumor properties of Scutellaria Barbata, especially in cervical cancer treatment(11). Antitumor and antiviral effects of Scutellaria Barbata are gaining attention in modern medicine and are of great significance in revealing the mechanism of anti-tumor effects and screening new anti-tumor drugs(12). Studies have shown that the main medicinal active components of Scutellaria Barbata are Baicalin and Wogonin(13). Baicalin and Wogonin were isolated and purified from Scutellaria Barbata by high performance liquid chromatography (HPLC). These two effective components did not contain endotoxin. It is difficult to elucidate the metabolic pathways and molecular mechanisms of TCM in vivo because of its many

components and complex metabolic processes[14]. Therefore, finding a research model that follows the TCM theory of starting from the whole and treating with evidence, which is in line with the requirements of TCM theory and prescription, and establishing a research model with multiple component, target and pathway action characteristics is the key to the modernization of TCM research[15]. With the rapid development of systems biology and computer technology network pharmacology came into being, network pharmacology is a new way of thinking about drug design based on network biology and multidirectional pharmacology, which is consistent with the view that TCM diagnoses and treats diseases from a holistic perspective and TCM regulates diseases from a holistic level[16]. Therefore, we mainly focused on network pharmacology analyses of Scutellaria Barbata as a potential treatment for cervical cancer.

2. Material and methods

2.1 Prediction the mechanism of action of Scutellaria Barbata on cervical cancer by network pharmacology

2.1.1 The chemical composition of Scutellaria Barbata collection and screening

The potential active components of Scutellaria Barbata were screened by TCMSP (https://old. tcmsp-e.com/tcmsp.php), a systematic pharmacology platform for Chinese herbal medicines, and STITCH (https://stitch.embl.de/) database for chemical-protein interactions, and the chemical formulas were constructed. Oral bioavailability (OB) ≥30% and drug-like properties (DL) ≥0.18 in the formulation of Hemerocallis were used to screen the active compounds. Component target prediction of hemicrania was performed by Swiss Target Prediction (http://www.swisstargetprediction.ch) and Drug Target Database (DrugBank, https://www.drugbank.com/) (Figure 1).

2.1.2 Acquisition of targets of action in cervical cancer

All known and predicted human genes in the genome, proteome, transcription, genetics, and function are available from the human Gene-disease-related database: OTP(https://www.open targets platform.org/), GeneCards(https://www.genecards.org/). This study was searched using the GeneCards database and the OTP database. Combine the results of the two databases, organize, merge and remove weight, and set the threshold to obtain the targets of cervical cancer disease.

2.1.3 Construction of a drug-active ingredient-target gene-disease network

Establish drug and disease signal association: the target genes corresponding to the active component of Scutellaria Barbata and the target genes related to cervical cancer are matched, and the common gene of the two is the key target of Scutellaria Barbata against cervical cancer. The Cytoscape3.7.2 software was used to construct the "drug-active ingredient-target gene-disease" relationship network to explore the mechanism of action against cervical cancer.

2.1.4 Construction of the relevant action network of key target proteins and the screening of core targets

The common genes obtained above were analyzed using the String platform (https://string-db.org/Version 10.5) database, and then the protein-protein interaction network (Protein-protein interaction, PPI) was constructed. PPI searches between known proteins and predicted proteins. The common target of Scutellaria Barbata and cervical cancer was entered into STRING11.0 (https://stringdb.org/) database, the data screening condition was limited to "multiple proteins", the species was set to "human", the placement level was 0.95, free nodes were excluded, and the other parameters were default values. The PPI network of target site was built, the results were saved as TSV file, and the saved results were imported into Cytoscape.3.7. 2 Software to obtain

the two highly interacting mechanism clusters. Topological analysis of the network was performed using the Network Analyzer plugin to screen out the top 10 core targets for cervical cancer treatment based on the degree (degree) values. Univariate survival analysis of the selected target genes was performed one by one, and the core gene targets highly correlated with the survival rate of cervical cancer patients were selected at $P < 0.1$.

2.1.5 GO functional enrichment analysis and KEGG pathway analysis

Gene ontology (gene ontology, GO) functional enrichment analysis and Kyoto Encyclopedia (Kyoto Encyclopedia of Genes and Genomes, KEGG) pathway analysis can identify key genes related to the prognosis of cervical cancer for further experimental studies. The 59 intersection genes were imported into the DAVID 6.8 database for GO functional enrichment analysis and KEGG pathway analysis. The role of the target of cervical cancer in GO function enrichment was analyzed from three modules: biological processes, molecular function and cellular components. Entries with $P < 0.05$ were selected for bars using GraphPad Prism 8.3.0 and KEGG pathway bubble plots using R software. The "core compound-target-pathway" network was constructed using Cytoscape 3.7.2 software. In the network graph, each node represents different compounds, targets or pathways, and the edges represent the interaction relationships between compounds, targets or pathways.

2.2 Experimental cell-level validation and action pathway validation of anti-cervical cancer mechanism

2.2.1 Cell Culture

Human cervical cancer cells (Hela) were presented by Department of Oncology, Affiliated Hospital of Southwest Medical University. Cells were cultured at 37°C, 5% CO_2 and 95% air in a medium containing 90%DMEM medium(Thermo Fisher , USA), 10% fetal bovine serum(FBS;

Hyclone)(Thermo Fisher, USA), and 1% penicillin-streptomycin. Control group: Hela cells + complete culture medium and experimental group corresponding time; Experimental group: Hela cells were treated with +0, 0.25, 0.5, 1, 2, 5, 10 mg/mL Baicalin (Sigma-Aldrich，USA) or Wogonin (Sigma-Aldrich，USA)for 0, 24, 48, 72 hours.

2.2.2 Assay for Cell Viability

A 96-well plate was seeded overnight with HeLa cells. HeLa cells were treated with concentration gradient of Baicalin (0, 0.25, 0.5, 1, 2, 5, 10 mg/mL), or the concentration gradient of Wogonin (0, 0.25, 0.5, 1, 2, 5, 10 mg/mL), the intervention time set for 0, 24, 48, 72 hours. In the following step, cell viability was measured using a Microplate Reader by using Cell Counting Kit8 (CCK-8).

2.2.3 Transwell Migration

According to the manufacturer's protocol, 24-well, 8.0 micron pore membranes (Corning, USA) were used. In the upper chamber, 1×10^5 cells were seeded in 200 litres of serum-free medium, and a chemoattractant of 800 liters of complete medium was added simultaneously to the lower chamber. Using cotton swabs, we removed the cells remaining on top of the membrane after incubation at 37°C for 24 hours, and the cells on the lower surface were those that had migrated. The cells that passed through the filter were photographed by inverted fluorescence microscope (Olympus, Japan) after being fixed with 4% paraformaldehyde and stained with 0.1% crystal violet solution.

2.2.4 TUNEL assay

In addition to the TUNEL assay, we measured apoptosis in cultured Hela cells fixed by 4% paraformaldehyde on coverslips. Under fluorescence microscopy, apoptotic Hela cells were observed using an in situ cell death detection kit (Corning Incorporated, China).

2.2.5 qRT-PCR

Invitrogen TRIzol reagent was used to extract total RNA from cultured cells, and 1 μg of total RNA was synthesized into cDNA.Target gene expression levels were assessed using SYBR R Premix Ex TaqTM II (Takara, Japan) followed by GAPDH normalization. Detailed primer sequences can be found in Supplementary Table S1.

2.2.6 Western Blot

10% SDS PAGE was used to separate the total protein samples and PVDF membranes were used as the transfer medium. 5% nonfat milk was used to block membranes, followed by primary antibodies and secondary antibodies that were HRP-tagged. Samples were quantified using Western blotting detection kit ECL Prime (GE Healthcare, USA) after culturing in TBST solution.

2.2.7 Statistical Analysis and Mapping

We analyzed the data using SPSS 25.0 and provided the results in the following form : mean ± standard deviation($\overline{X}$ ±s); The difference between the two groups was compared using a T-test, as One-way ANOVA indicated that there was a statistical difference between groups at $P<0.05$.

3. Results

3.1 Results of the network pharmacology analysis

3.1.1 Chemical composition and target information

A total of 14 active components were obtained and chemical formulas were constructed (Figure 2 and Table 1). 222 predicted targets were obtained by Swiss Target Prediction and the drug target database DrugBank.

Table 1 Composition of Scutellaria Barbata

	Compound	Formulas	Molecular Weight
1	quercretin-3-O-β-D-glucopyranside	$C_{21}H_{20}O_{12}$	464.10
2	luteilin-7-O-B-D-glucopyranside	$C_{21}H_{20}O_{11}$	448.10
3	rutin	$C_{27}H_{30}O_{16}$	610.15
4	apigenin-7-O-B-D-glucopyranoside	$C_{21}H_{20}O_{10}$	432.11
5	hispidulin-7-O- β-D-glucuronide	$C_{22}H_{20}O_{12}$	476.10
6	baicalin	$C_{21}H_{18}O_{11}$	446.08
7	scutellarein	$C_{15}H_{10}O_{6}$	286.05
8	luteolin	$C_{15}H_{10}O_{6}$	286.05
9	quercetin	$C_{15}H_{10}O_{7}$	302.04
10	apigenin	$C_{15}H_{10}O_{5}$	270.05
11	naringenin	$C_{15}H_{12}O_{5}$	272.07
12	baicalein	$C_{15}H_{12}O_{5}$	272.07
13	wogonin	$C_{16}H_{12}O_{5}$	284.07
14	chrysin	$C_{15}H_{10}O_{4}$	254.06

3.1.2 Related targets of cervical cance

761 cervical cancer-related targets were obtained from the GeneCards and OTP databases. Using 222 predicted targets and 761 related targets of cervical cancer, the venney 2.1.0 online analysis tool was used to draw a visual Venn diagram of venney 2.1.0,59 intersection target genes were obtained.(Figure 3).

3.1.3 Construction of Scutellaria Barbata-active ingredient-targets gene-cervical cancer

network

Target genes corresponding to Scutellaria Barbata's active ingredient and the target genes related to cervical cancer were compared for drug-disease associations, and the common genes between them were obtained as the key targets of Scutellaria Barbata against cervical cancer. Cytoscape 3.7.2 was used to construct the "Scutellaria Barbata-active ingredient-targets gene-cervical cancer" network to investigate the mechanism of action of Scutellaria Barbata against cervical cancer(Figure 4).

3.1.4 Protein Interaction Network and Access to Core Targets

The String platform was used to search for known and predicted protein interactions. The common targets of Scutellaria Barbata and cervical cancer were entered into the String database to obtain 27 core targets (Figure 5 and Table 2). Univariate survival analysis analyzed the 27 core targets, and the core gene targets were highly associated with the survival of cervical cancer patients: EGFR, MDM2, TNF and VEGFA (Figure 6).

Table 2 Information of core targets

Number	Gene Symbol	Uniprot ID	Protein Name	OS-p(HR)	Degree
1	EGFR	P00533	Epidermal growth factor receptor erbB1	0.056	49
2	VEGFA	P15692	Vascular endothelial growth factor A	0.032	48
3	AKT1	P31749	Serine/threonine-protein kinase AKT	0.63	47
4	ESR1	P03372	Estrogen receptor alpha	0.76	45
5	SRC	P12931	Tyrosine-protein kinase SRC	0.93	44
6	BCL2L1	Q07817	Apoptosis regulator Bcl-X	0.94	40
7	TNF	P01375	TNF-alpha	0.067	39
8	MDM2	Q00987	E3 ubiquitin-protein ligase Mdm2	0.09	39
9	PIK3CA	P42336	PI3-kinase p110-alpha subunit	0.96	35
10	PTGS2	P35354	Cyclooxygenase-2	0.16	31
11	MMP9	P14780	Matrix metalloproteinase 9	0.41	31
12	AR	P10275	Androgen Receptor	0.44	30
13	KDR	P35968	Vascular endothelial growth factor receptor 2	0.91	28
14	KIT	P10721	Stem cell growth factor receptor	0.53	28
15	PGR	P06401	Progesterone receptor	0.22	28
16	IL2	P60568	Interleukin-2	0.42	28
17	MCL1	Q07820	Induced myeloid leukemia cell differentiation protein Mcl-1	0.44	28
18	PIK3R1	P27986	PI3-kinase p85-alpha subunit	0.9	28
19	MMP2	P08253	Matrix metalloproteinase 2	0.41	27
20	IGF1R	P08069	Insulin-like growth factor I receptor	0.31	26

21	CDK4	P11802	Cyclin-dependent kinase 4	0.12	26
22	CDK2	P24941	Cyclin-dependent kinase 2	0.19	26
23	MET	P08581	Hepatocyte growth factor receptor	0.42	25
24	CASP9	P55211	Caspase-9	0.61	24
25	PTPRC	P08575	Receptor-type tyrosine-protein phosphatase C	0.29	24
26	PPARG	P37231	Peroxisome proliferator-activated receptor gamma	0.23	24
27	PARP1	P09874	Poly (12) polymerase-1	0.42	24

3.1.5 GO functional enrichment analysis and KEGG pathway enrichment analysis

DAVID6.8 was used to analyze GO functional enrichment of 59 intersecting genes, and the top 10 CC ranked entries were plotted in a bar graph after sorting the number of gene enrichment Count with $P<0.05$. Cellular responses to chemical stimuli, organic substances, and positive regulation of cellular metabolic processes were the main biological processes enriched. From the aspect of cellular components, the main enrichment is in extracellular region, extracellular region part, cytosol, etc. In terms of molecular function, the main enrichment is in organic cyclic compound binding, enzyme binding, receptor binding, protein, etc(Figure 7). DAVID6.8 was used to analyze KEGG pathway in a bar graph after sorting the number of gene enrichment, and 79 signaling pathways were obtained. After screening with $P<0.05$, R software was used to perform KEGG pathway enrichment analysis on 11 signaling pathways, including pathways involved in cancer, TNF signaling pathways, and the other pathways(Figure 8).

3.2 The results of cell level experiments and pathway verification of the anti-cervical cancer mechanism of Scutellaria Barbata

3.2.1 The anti-proliferative and anti-migratory activity of Baicalin and Wogonin was observed

in cervical cancer cells

Cell validation experiments confirmed that Baicalin and Wogonin could effectively inhibit the proliferation of Hela cells: CCK 8 test found that the proliferation rate of the experimental group decreased significantly at 48 h and 72 h compared with the control group (P <0.001), and the most obvious when the drug concentration was 10 mg/mL (P <0.001). Within 72h, as time passed and the drug concentration increased (P <0.01). Inhibition of Hela cell proliferation was more pronounced at drug concentrations of 5 mg/mL and 10 mg/mL (Figure 9A, B). Transwell Migration assay The detection of cells in the experimental and control groups showed that the migration ability of the experimental group was significantly weaker compared with that of the control group. At the same time point, after the addition of baicalin and baicalein, the migration inhibition effect was more obvious than that of the single agent (P <0.001, Figure 9C).

3.2.2 Baicalin and Wogonin promote Hela cells apoptosis

The results of Tunel staining method showed the highest apoptosis ratio to Hela cells after 72 h of single agent Baicalin or Wogonin with 10 mg/mL. Baicalealin and Wogonin were respectively cultured in (10 mg/mL + 10 mg/mL) for 12 h, and the apoptosis ratio in the double drug group was higher than that in the single drug group. As the drug concentration increased, the apoptosis ratio increased more significantly in the high dose group than in the corresponding low dose group, and the difference between the single and double drug groups and the corresponding low dose group was statistically significant (P <0.001) (Table 3, Figure 10).

Table 3 Apoptosis rate (mean ± SD)

group	0mg/mL Baicalin	5mg/mL Baicalin	10mg/mL Baicalin
0mg/mL Wogonin	0.10±0.02	0.20±0.03[(1)]	0.45±0.07[(1)(3)]

5mg/mL Wogonin	0.38±0.04(1)	0.46±0.11(1)	-
10mg/mL Wogonin	0.58±0.03(1)	-	0.65±0.06(1)(4)

3.2.3 Pathway validation of the effects of Baicalealin and Wogonin in Hela cells

The mRNA of EGFR, TNF- α and VEGFA, PI3K and AKT, and MDM 2 gene were detected for 72 h after culture of different experimental and control Hela cells by RT-PCR, and the protein expression was determined by Western blot. The results showed that the mRNA corresponding to the control group, TNF- α, VEGFA, the mRNA (the Figure 11A, B), and protein expression, Figure 11C, D), and with the increase of the drug concentration, the decrease was more obvious. Moreover, under the same drug dose condition, the effect of Wogonin group was more obvious than Baicalin, and the effect of double drug group was more obvious than that of single drug group.

3.2.4 Potential mechanisms of Baicalin and Wogonin against cervical cancer

PI3K-AKT signaling pathway is a classical signaling pathway in cells. PI3K is a proto-oncogene whose chemical function can phosphorylate phospholithphthalinositol and its derivatives to generate phospholithphthalinositol 3,4, 5 one triphosphate (PIP3). Epithelial interstitial transformation (EMT) is a biological process in which epithelial cells are transformed into cells with a stromal phenotype through specific procedures. EMT enables tumor cells to acquire the ability to infiltrate and transfer into surrounding tissues. PIP3 Bring domain proteins such as AKT 1 and PDPK 1 to the cell membrane, downregulate the expression of MDM 2 gene and then inhibit the effect of epithelial-mesenchymal transition, thus activating the signaling cascade of cell growth, proliferation, motility and morphology at the corresponding level to play a key role. As shown in the above results, according to the prediction of network pharmacology, EGFR, TNF- α, VEGFA, PI3K, AKT were selected as the study indicators, confirming that the mechanism of anti-cervical proliferation,

migration and apoptosis may be related to the inhibition of PI3K-Akt signaling, and then the inhibition of epithelial-mesenchymal transformation by inhibiting MDM 2 expression. The progression of cervical cancer and chemotherapy resistance are also closely associated with the overactivation of PI3K-AKT pathway, which can exacerbate the proliferation and migration of cervical cancer cells and inhibit the apoptosis of cervical cancer cells. After combining with the EGFR, TNF-α and VEGFA hub gene targets of cervical cancer, the active ingredients of Scutellaria Barbata were involved in the regulation of PI3K-AKT signaling pathway and the down-regulation of the expression of MDM2 genes and thus inhibited the epithelial-mesenchymal transition, which effectively inhibited the proliferation, migration and promoted the apoptosis of cervical cancer cells (Figure 12).

4. Discussion

The Chinese herb Scutellaria Barbata is the dried whole herb of Scutellaria Barbata, which is a purgative and detoxifying traditional Chinese medicine, commonly used clinically for the treatment of primary liver cancer, gastrointestinal tumors, nasopharyngeal cancer and breast cancer, and externally for the treatment of painful boils and bruise[17, 18]. Chemical composition studies showed that the main active ingredients of Scutellaria Barbata are quercetin, lignan, baicalin, wogonin, etc. Polysaccharides, volatile oils, flavonoids, and diterpenoids are the main chemical components[19]. Pharmacological studies have shown that Scutellaria Barbata has pharmacological activities such as antitumor, antioxidant, antibacterial and immunomodulatory[20, 21]. In this study, using network pharmacology, we found that 14 chemical components may be the active components of Scutellaria Barbata against cervical cancer, with 222 targets related to these 14 major components. Through the cervical cancer disease target mapping, found that 761 targets, intersection of 59, the key target PPI

network topological characteristics analysis found 27 core targets, by "Scutellaria Barbata-components-target-cervical cancer" network visible components and target of one-to-many complex relationship, it also just verified the Scutellaria Barbata components, multiple target characteristics. GO and KEGG enrichment analysis of key targets predicted the cancer pathway, PI3K-AKT-MDM2 expression pathway, VEGFA signaling pathway, and TNF signaling pathway for cervical cancer.

Limited to network pharmacology, the stock of data for traditional Chinese medicine research is small, and some data are one-sided, especially the data related to Scutellaria Barbata, which leads to the limitation of prediction results. Therefore, necessary biological experiments are needed to further verify the relevant mechanisms of network pharmacology prediction and mining.

Scutellaria Barbata has shown great potential in antitumor treatments, particularly for cervical cancer in recent years[22]. The antitumor and antiviral effects of Chinese medicine are increasingly attracting the attention of modern medicine, which is important for revealing the mechanism of antitumor effects of Chinese medicine more deeply and screening new antitumor drugs[23]. In order to improve the cure rate and survival rate, in addition to striving to eradicate the disease and improve the survival rate, the treatment should also strive to preserve the reproductive endocrine function of patients when possible, especially for young patients[24]. Flavonoids have good antitumor activity[25]. Quercetin, baicalin, lignan, and apigenin can promote apoptosis of cervical cancer cells and inhibit tumor proliferation and invasion[13]. Various pharmacological activities of Saccharomyces cerevisiae have been discovered in plants such as antioxidant, anti-inflammatory, antitumor, and neuroprotective: saccharomyces cerevisiae prevents 1,2-dimethylhydrazine-induced experimental colon cancer in male albino Wistar rats; saccharomyces cerevisiae inhibits the transformation of

mouse epithelial cells JB6C141 to cancer cells. Gallopanaxanthin inhibited colon cancer cells[26, 27] . It has been shown that baicalein can inhibit HeLa cell proliferation and invasion, and its inhibitory effect may be related to down-regulation of COX-2 and VEGF protein expression[28]. In addition, Baicalein exerted a good inhibitory effect on the proliferation and migration invasion of MDA-MB-231 and MCF-7 human breast cancer cells at the in vitro cellular level, and exerted its oncogenic effect through the classical Wnt/β-catenin signaling pathway[29-31]. Studies have shown that the p38/JNK/ERK pathway can inhibit the proliferation, migration and invasion of cervical cancer cells and promote the apoptosis of cervical cancer cells[32, 33].

Two common active components of Baicalealin and Wogonin were selected from the 14 active components to further verify the results of network pharmacology analysis[13]. Hela cells were selected as the experimental subjects in vitro. The stimulation concentrations of Baicalealin and Wogonin were set to 0, 0.25, 0.5, 1, 2, 5 and 10 mg/mL, and the proliferation of Hela cells was detected at 0, 24, 48 and 72 h after stimulation, respectively. We found that Baicalealin and Wogonin had an inhibitory effect on the proliferation of Hela cells, and with the increase of drug concentration and stimulation time, the inhibitory effect was more obvious than that of the control group, and the inhibitory effect of the two active ingredients on the growth of Hela cells was concentration - and time-dependent. According to the results of CCK8 test, we selected 5 and 10 mg/mL concentration as the stimulation concentration, and 48 and 72 h as the stimulation time to continue to detect the effects of Baicalealin and Wogonin on the migration and apoptosis of Hela cells. The results showed that Baicalealin and Wogonin could significantly inhibit the migration of Hela cells, and when the two active ingredients were stimulated at the same time, the inhibitory effect was more obvious than that of single drug stimulation, but there was no statistically significant difference in the inhibitory

efficiency of migration between the 5 mg and 10 mg concentration groups. When 5 mg and 10 mg Baicalin and/or Wogonin were used to stimulate Hela cells for 72 h, we found that compared with the control group, the apoptosis rate in the drug stimulation group was significantly increased, and the number of apoptotic cells in the high concentration group was significantly higher than that in the low concentration group, and the apoptosis rate was also higher when the two drugs were simultaneously stimulated than when the single drug was stimulated. Therefore, we believe that Baicalealin and Wogonin can effectively inhibit the proliferation and migration of cervical cancer cells, and enhance the apoptosis of cells. Some of the targets with strong docking ability in this study, such as EGFR, MDM2, TNF, and VEGFA, were clearly linked to cervical carcinogenesis and development. Cytochrome P450 is involved in a variety of human metabolic functions, and the genetic polypeptide nature of MDM2 may play a role in the occurrence and development of cervical cancer(34). TNF and VEGFA are more commonly seen in studies of drugs that promote and inhibit sex hormone secretion, and EGFR inhibits gonadal axis secretion in breast cancer patients(35). EGFR levels are not associated with the risk of death and recurrence of cervical cancer, suggesting EGFR is not related to the mechanism of cervical carcinogenesis and progression(36). Compound activity is closely related to pharmacokinetic properties, suggesting that molecular docking can only be an effective means of screening active ingredients, and subsequent in vivo and ex vivo experiments are still needed for validation(37). In recent years, there have been remarkable achievements in TCM research on gynecological diseases, but there are still many scientific challenges that need to be solved(38). In this study, we aimed to investigate the molecular mechanism of action of the active chemical components of Scutellaria Barbata on the VEGF involved pathway in cervical cancer through systematic pharmacological data mining, and to provide a strong basis for subsequent drug

development or cervical cancer treatment[39]. Existing findings have been elaborated for cervical cancer VEGF expression mechanisms and drug mechanism of action studies, but not in depth, in a retrospective cohort study of 64 patients, protein expression was obtained by immunohistochemistry from biopsies containing tumors and stroma[40]. It has been demonstrated that miR-125 as affects a variety of cancer-related and to expression, which is downregulated in cervical cancer tissues and cell lines, while VEGF is upregulated[41]. Negatively regulates VEGF expression in cervical cancer tissues by targeting and regulating VEGF to inhibit proliferation, invasion and migration in cervical cancer[42]. It was demonstrated that miR-520d-5p inhibited the activation of PI3K-AKT signal pathway, thereby suppressing the progression and development of cervical cancer[41]. Numerous studies have shown that, EGFR is an oncogene in cervical cancer, The rise of EGFR is closely related to the occurrence and development of cervical cancer, While our findings suggest that, Most likely, by inhibiting EGFR expression, Thus to play a role in the treatment of cervical cancer, And suggested in the KEGG pathway enrichment analysis, The PI3K-AKT-related pathway may be an important pathway affecting the development of cervical cancer, In combination with the preexisting studies, It cannot be excluded that by inhibiting the expression of EGFR, Affect the PI3K-AKT related pathway and thus decrease the proliferation and migration of cervical cancer cells, Thus to play a therapeutic role in cervical cancer.

Tumor necrosis factor (TNF),as a member of the TNF superfamily of cytokines, mediates the processes of cellular inflammation, differentiation, proliferation and apoptosis[43]. And the current reference to TNF generally refers to TNF-α accounting for about 70%-95% of the total TNF bioactivity[44].TNF plays different regulatory roles in different diseases such as coronary heart disease, rheumatoid arthritis, cancer, and ankylosing spondylitis, and especially its anti-tumor effects

have received much attention in recent years. It has been found that the malignancy of cervical cancer is positively correlated with cellular TNF expression, and therefore, cellular TNF-α expression can be used as an observational index for cervical cancer grading as well as prognosis(45). TNF-α plays an important role in the induction of cervical cancer cell proliferation, and it has also been demonstrated that TNF-α is correlated with cervical cancer in the elderly.The TNF signal pathway is an important inflammatory response signal pathway, in which the associated factor receptors can also induce cellular regulation(46). The MAPK signal pathway is one of the classical signal pathways, and studies have shown that this pathway promotes cervical cancer angiogenesis by regulating the expression of VEGF vascular-related factors, and conversely, blocking this pathway inhibits the proliferation and angiogenesis of cervical cancer cells(47). In our in vitro validation experiment, we verified the potential targets, pathways and target genes of the above active components. The results showed that both of the two active components stimulated little and could decrease the mRNA and protein expressions of the three possible targets of EGFR, TNF-α and VEGFA in Hela cells. The expression of two signal pathway proteins and their downstream target genes, which were speculated by network pharmacology, were also decreased, suggesting that the active components of Scutellaria Barbata were likely to regulate the PI3K-AKT signaling pathway by acting on the three targets of EGFR, TNF-α and VEGFA, and down-regulating the expression of MDM2 gene. Therefore, the proliferation and migration of Hela cells were inhibited and the apoptosis of Hela cells was enhanced.

5. Conclusion

In conclusion, the effective components of the Scutellaria Barbata mainly treat cervical cancer through multiple components, multiple targets, multiple pathway, EGFR, TNF-α, VEGFA are the key

targets related with the prognosis of cervical cancer, and the effective components of the Scutellaria Barbata can, by regulating the PI3K-AKT signaling pathway, inhibit epithelial-mesenchymal transition, thus effectively inhibit the proliferation, migration and promote apoptosis, provide reference for further research.

6. Acknowledgments

We are grateful for TCMSP/STITCH/DisGeNET websites for providing access to the data. Thanks to GeneCard database and DrugBank database for free use.

7. References

1. Hu Z, Ma D. The precision prevention and therapy of HPV-related cervical cancer: new concepts and clinical implications. Cancer medicine. 2018;7(10):5217-36.

2. Shen S, Zhang S, Liu P, Wang J, Du H. Potential role of microRNAs in the treatment and diagnosis of cervical cancer. Cancer genetics. 2020:25-30.

3. Feng CH, Mell LK, Sharabi AB, McHale M, Mayadev JS. Immunotherapy With Radiotherapy and Chemoradiotherapy for Cervical Cancer. Semin Radiat Oncol. 2020;30(4):273-80.

4. Stevanović S, Draper L, Langhan M, Campbell T, Kwong M, Wunderlich J, et al. Complete regression of metastatic cervical cancer after treatment with human papillomavirus-targeted tumor-infiltrating T cells. Journal of clinical oncology : official journal of the American Society of Clinical Oncology. 2015;33(14):1543-50.

5. Kagabu M, Nagasawa T, Sato C, Fukagawa Y, Kawamura H, Tomabechi H, et al. Immunotherapy for Uterine Cervical Cancer Using Checkpoint Inhibitors: Future Directions. International journal of molecular sciences. 2020;21(7).

6. Randall LM, Walker AJ, Jia AY, Miller DT, Zamarin D. Expanding Our Impact in Cervical Cancer Treatment: Novel Immunotherapies, Radiation Innovations, and Consideration of Rare Histologies. Am Soc Clin Oncol Educ Book.

2021;41:252-63.

7. Morris G, Lim-Wilby M. Molecular docking. Methods in molecular biology (Clifton, NJ). 2008;443:365-82.

8. Saikia S, Bordoloi M. Molecular Docking: Challenges, Advances and its Use in Drug Discovery Perspective. Current drug targets. 2019;20(5):501-21.

9. Zhang W, Chao X, Wu J, Ma X, Yang Y, Wu Y, et al. Exploring the Potential Mechanism of Guchang Zhixie Wan for Treating Ulcerative Colitis by Comprehensive Network Pharmacological Approaches and Molecular Docking Validation as Well as Cell Experiments. Chemistry & biodiversity. 2021;18(1):e2000810.

10. Gaillard T. Evaluation of AutoDock and AutoDock Vina on the CASF-2013 Benchmark. Journal of chemical information and modeling. 2018;58(8):1697-706.

11. Balkwill F. Cancer and the chemokine network. Nature reviews Cancer. 2004;4(7):540-50.

12. Brat D, Bellail A, Van Meir E. The role of interleukin-8 and its receptors in gliomagenesis and tumoral angiogenesis. Neuro-oncology. 2005;7(2):122-33.

13. Wang Q, Xu H, Zhao X. Baicalin Inhibits Human Cervical Cancer Cells by Suppressing Protein Kinase C/Signal Transducer and Activator of Transcription (PKC/STAT3) Signaling Pathway. Medical science monitor : international medical journal of experimental and clinical research. 2018;24:1955-61.

14. Yan R, Shuai H, Luo X, Wang X, Guan B. The clinical and prognostic value of CXCL8 in cervical carcinoma patients: immunohistochemical analysis. Bioscience reports. 2017;37(5).

15. Wang J, Zheng H, Han Y, Wang G, Li Y. A Novel Four-Gene Prognostic Signature as a Risk Biomarker in Cervical Cancer. International journal of genomics. 2020;2020:4535820.

16. Tian R, Li X, Gao Y, Li Y, Yang P, Wang K. Identification and validation of the role of matrix metalloproteinase-1 in cervical cancer. International journal of oncology. 2018;52(4):1198-208.

17. Khan S, Kamal M. Wogonin Alleviates Hyperglycemia Through Increased Glucose Entry into Cells Via AKT/GLUT4

Pathway. Current pharmaceutical design. 2019;25(23):2602-6.

18. Yao H, Yan C, Qiumin H, Li Z, Jiao A, Xin L, et al. Epidemiological Trends and Attributable Risk Burden of Cervical Cancer: An Observational Study from 1990 to 2019. International journal of clinical practice. 2022;2022:3356431.

19. Pan L, Cho K, Yi I, To C, Chen D, Do C. Baicalein, Baicalin, and Wogonin: Protective Effects against Ischemia-Induced Neurodegeneration in the Brain and Retina. Oxidative medicine and cellular longevity. 2021;2021:8377362. doi:10.1155/2021/8377362.

20. Siegel R, Miller K, Fuchs H, Jemal A. Cancer statistics, 2022. CA: a cancer journal for clinicians. 2022;72(1):7-33. doi:10.3322/caac.21708.

21. Yang X, Zheng S, Wang X, Wang J, Ali Shah S, Wang Y et al. ScutellariaAdvances in pharmacology, biosynthesis, and metabolic engineering of -specialized metabolites. Critical reviews in biotechnology. 2022:1-17. doi:10.1080/07388551.2022.2149386.

22. Huang Z, Yang Y, Fan X, Ma W. Network pharmacology-based investigation and experimental validation of the mechanism of scutellarin in the treatment of acute myeloid leukemia. Frontiers in pharmacology. 2022;13:952677.

23. Banik K, Khatoon E, Harsha C, Rana V, Parama D, Thakur K, et al. Wogonin and its analogs for the prevention and treatment of cancer: A systematic review. Phytotherapy research : PTR. 2022;36(5):1854-83.

24. Xue S, Geng A, Lian T, Liu Y. Scutellaria barbata D. Don inhibits cervical cancer cell proliferation, migration, and invasion via miR-195-5p/LOXL2 axis. Toxicology research. 2022;11(5):804-11.

25. Kashafi E, Moradzadeh M, Mohamadkhani A, Erfanian S. Kaempferol increases apoptosis in human cervical cancer HeLa cells via PI3K/AKT and telomerase pathways. Biomedicine & pharmacotherapy = Biomedecine & pharmacotherapie. 2017;89:573-7.

26. Carrillo L, Seidman D, Cittadini E, Meirow D. The role of fertility preservation in patients with endometriosis. Journal of assisted reproduction and genetics. 2016;33(3):317-23.

27. Chen J, Teng J, Ma L, Tong H, Ren B, Wang L, et al. Limonium bicolorFlavonoids Isolated From the Flowers of and their Antitumor Evaluation. Pharmacognosy magazine. 2017;13(50):222-5.

28. Chen R, Yang W, Li Y, Cheng X, Nie Y, Liu D, et al. Effect of immunotherapy on the immune microenvironment in advanced recurrent cervical cancer. International immunopharmacology. 2022;106:108630.

29. Sun P, Wang A, Zhang Z, Zhang Y, Zheng X. Network pharmacology-based strategy to investigate the active ingredients and molecular mechanisms of Scutellaria Barbata D. Don against radiation pneumonitis. Medicine. 2021;100(47):e27957.

30. Tang T, Li S, Pan B, Xiao J, Pang Y, Xie S, et al. Scutellaria barbataIdentification of Flavonoids from D. Don as Inhibitors of HIV-1 and Cathepsin L Proteases and Their Structure-Activity Relationships. Molecules (Basel, Switzerland). 2023;28(11).

31. Li K, Pan W, Ma Y, Xu X, Gao Y, He Y, et al. BMX activates Wnt/β-catenin signaling pathway to promote cell proliferation and migration in breast cancer. Breast cancer (Tokyo, Japan). 2020;27(3):363-71.

32. Zhou P, Liu P, Zhang J. Long noncoding RNA RUSC1‐ AS‐ N promotes cell proliferation and metastasis through Wnt/β‐ catenin signaling in human breast cancer. Molecular medicine reports. 2019;19(2):861-8.

33. Hu J, Zhang Y, Guo P, Tang N, Lu Y, Jin Z. Taraxastane inhibits the proliferation, migration and invasion of human cervical cancer by inducing ROS- mediated necrosis like cell death, cell cycle arrest and modulation of JNK/MAPK signaling pathway. Journal of BUON : official journal of the Balkan Union of Oncology. 2020;25(2):716-22.

34. Xin W, Zhang J, Zhang H, Ma X, Zhang Y, Li Y, et al. CLCA2 overexpression suppresses epithelial-to-mesenchymal transition in cervical cancer cells through inactivation of ERK/JNK/p38-MAPK signaling pathways. BMC molecular and cell biology. 2022;23(1):44.

35. Chou R, Hsieh S, Yu Y, Huang M, Huang Y, Hsieh Y. Fisetin inhibits migration and invasion of human cervical cancer cells by down-regulating urokinase plasminogen activator expression through suppressing the p38 MAPK-dependent

NF-κB signaling pathway. PloS one. 2013;8(8):e71983.

36. Zhang L, Ren B, Zhang J, Liu L, Liu J, Jiang G, et al. Anti-tumor effect of Scutellaria barbata D. Don extracts on ovarian cancer and its phytochemicals characterisation. J Ethnopharmacol. 2017;206:184-92.

37. Abdel-Aziz A, Gamal El-Tahawy N, Salah Abdel Haleem M, Mohammed M, Ali A, Ibrahim Y. Amelioration of testosterone-induced benign prostatic hyperplasia using febuxostat in rats: The role of VEGF/TGFβ and iNOS/COX-2. European journal of pharmacology. 2020;889:173631.

38. Jing XU, Feng-Qin S, Ke-Xin DU, Jia D, Dong-Yun LI. [Mechanism of "Scutellaria barbata-Hedyotis diffusa" against breast cancer based on network pharmacology]. Zhongguo Zhong Yao Za Zhi. 2020;45(18):4448-54.

39. Guo SS, Shi YJ, Gao YJ, Su D, Cui XL. [The cytology mechanism of anti-parainfluenza virus infection of total flavone of Scutellaria barbata]. Yao Xue Xue Bao. 2009;44(12):1348-52.

40. Franco P, Simões R, Carbonel A, Sasso G, Florencio-Silva R, Baracat E, et al. The influence of phytoestrogens or estrogens on the proliferation of the rat endocervical mucosa. Revista da Associacao Medica Brasileira (1992). 2020;66(2):174-9.

41. Johnson M, Mowa C. Transcriptomic profile of VEGF-regulated genes in human cervical epithelia. Cell and tissue research. 2021;384(3):771-88.

42. Azevedo Martins J, Rabelo-Santos S, do Amaral Westin M, Zeferino L. Tumoral and stromal expression of MMP-2, MMP-9, MMP-14, TIMP-1, TIMP-2, and VEGF-A in cervical cancer patient survival: a competing risk analysis. BMC cancer. 2020;20(1):660.

43. Fu K, Zhang L, Liu R, Shi Q, Li X, Wang M. MiR-125 inhibited cervical cancer progression by regulating VEGF and PI3K/AKT signaling pathway. World journal of surgical oncology. 2020;18(1):115.

44. Hemalatha KL, Stanely Mainzen Prince P. Anti-inflammatory and anti-thrombotic effects of zingerone in a rat model of myocardial infarction. Eur J Pharmacol. 2016;791:595-602.

45. Wu N, Yuan T, Yin Z, Yuan X, Sun J, Wu Z, et al. Network Pharmacology and Molecular Docking Study of the Chinese Miao Medicine Sidaxue in the Treatment of Rheumatoid Arthritis. Drug Des Devel Ther. 2022;16:435-66.

46. Cruceriu D, Baldasici O, Balacescu O, Berindan-Neagoe I. The dual role of tumor necrosis factor-alpha (TNF-α) in breast cancer: molecular insights and therapeutic approaches. Cell Oncol (Dordr). 2020;43(1):1-18.

47. Simões RT, Gonçalves MA, Donadi EA, Simões AL, Bettini JS, Duarte G, et al. Association of tumor necrosis factor a-2 and a-8 microsatellite alleles with human papillomavirus and squamous intraepithelial lesions among women in Brazil. J Clin Microbiol. 2005;43(8):3932-7.

48. Mbulaiteye S, Kemp T, Gage J, Ajenifuja K, Kiruthu C, Wentzensen N, et al. Plasma cytokine levels and human papillomavirus infection at the cervix in rural Nigerian women. Cytokine. 2013;64(1):146-51.

49. Tohyama A, Murakami M, Yoshino K. Strategies for the Treatment of Cervical Cancer with Bulky Pelvic Lymph Nodes: An Overview of the Current Evidence. Journal of UOEH. 2020;42(4):317-25.

8. Supporting information captions

We have resubmitted our manuscript and checked and uploaded all the raw data, codes, and images to the Jianguoyun. We hope you can easily find the source data through this link: https://www.jianguoyun.com/p/DbTgePYQ-NDvCxjDtZYFIAA.

9. Figure Legends

Figure 1 Flow chart of collection and screening of chemical components of Scutellaria Barbata

Figure 2 Chemical composition and target information of Chinese medicine

Figure 3 Scutellaria Barbata active compound target-disease related target visualization Venn diagram

Figure 4 Drug-active ingredient-target gene-disease network

Figure 5 Protein Interaction Network and Access to Core Targets

Figure 6 Core targets for single factor survival analysis: (A) The relationship between EGFR expression and overall survival of cervical cancer patients; (B) The relationship between VEGFA expression and overall survival of cervical cancer patients; (C) The relationship between TNF expression and overall survival rate of cervical cancer patients; (D) The relationship between MDM2 expression and overall survival in patients with cervical cancer.

Figure 7 GO function enrichment analysis

Figure 8 KEGG functional enrichment analysis

Figure 9 Detection of proliferation and migration of Hela cells: (A) The effects of different concentrations of Baicalin on Hela proliferation were detected by CCK8 at different time points; (B) The effect of different concentrations of Wogonin on Hela proliferation detected by CCK8 at different time points; (C) Transwell Migration assay to determine the effect of Baicalin and Wogonin on Hela cell migration at different concentrations.

Note: Compared with 48 h control group, P < 0.001; ***, P < 0.001 compared with 72 h control group; (1) Compared with control group at the same time point, P < 0.001; (2) Compared with the control group at the same time point, P < 0.01; (3) Compared with the control group at the same time point, P < 0.05; (4) Compared with Baicalin group with the same concentration at the same time point, P < 0.001; (5) Compared with Wogonin group with same concentration at the same time point, P < 0.001; (6) Compared with Wogonin group with the same concentration at the same time point, P < 0.05.

Figure 10 Detection of apoptosis of Hela cells. Note:(1) Compared with the control group, P < 0.001; (2) Compared with the control group at the same time point, P < 0.05; (3) Compared with Baicalin 5 mg group, P < 0.05; (4) Compared with Baicalin 10 mg group, P < 0.001.

Figure 11 mRNA and protein expression of target sites and pathways of effective components of Scutellaria Barbata:(A) mRNA expression of target sites after stimulation of Hela cells with different concentrations of Baicalin and/or Wogonin; (B) mRNA expression of the effect pathway after stimulation of Hela cells with different concentrations of Baicalin and/or Wogonin; (C) Expression of target protein after stimulation of Hela cells with different concentrations of Baicalin and/or Wogonin; (D) Expression of effector pathway protein after stimulation of Hela cells with different concentrations of Baicalin and/or Wogonin. Note: (1) Compared with the corresponding

control group, $P < 0.001$; (2) Compared with the corresponding control group, $P < 0.01$; (3) Compared with the corresponding control group, $P < 0.05$; (4) Compared with the corresponding control group, $P > 0.05$.

Figure 12 Targets and potential mechanisms of pathways for the action of effective components of Sculellaria Barbata annuus in cervical cancer

Figure 1

Click here to access/download;Figure;figure 1.tif

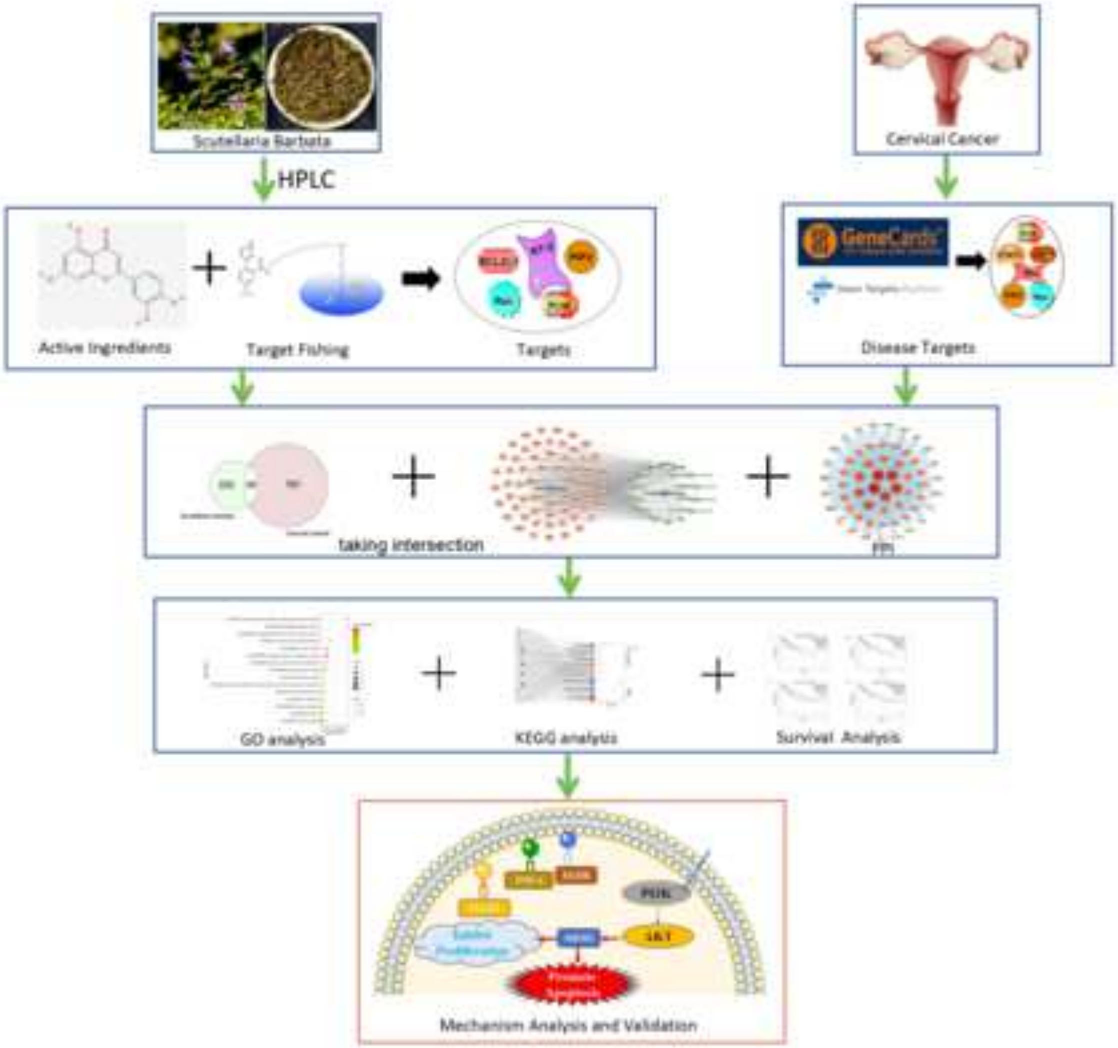

Figure 2

Click here to access/download;Figure;Figure 2.tiff

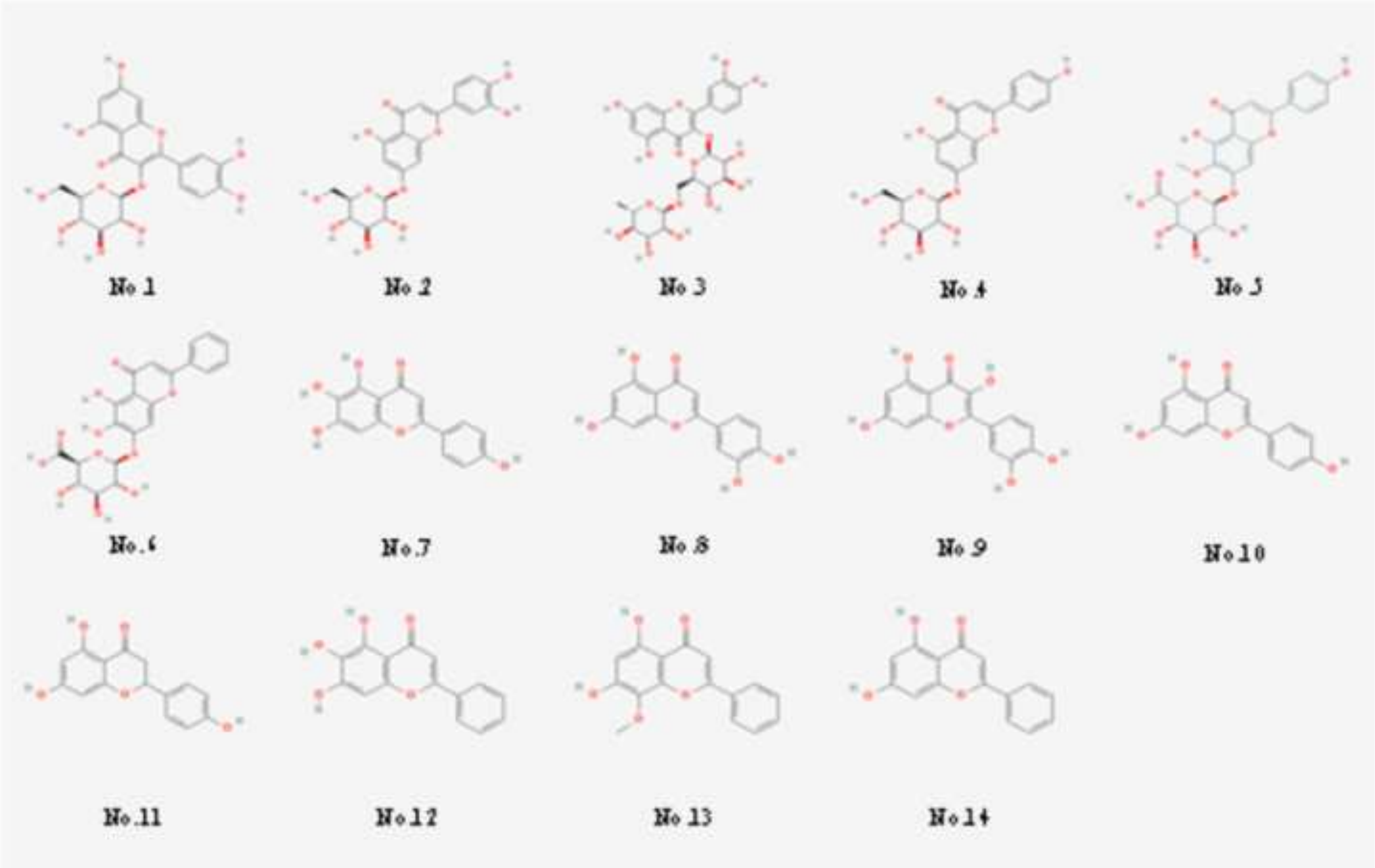

Figure 3

Click here to access/download;Figure;Figure 3.tiff

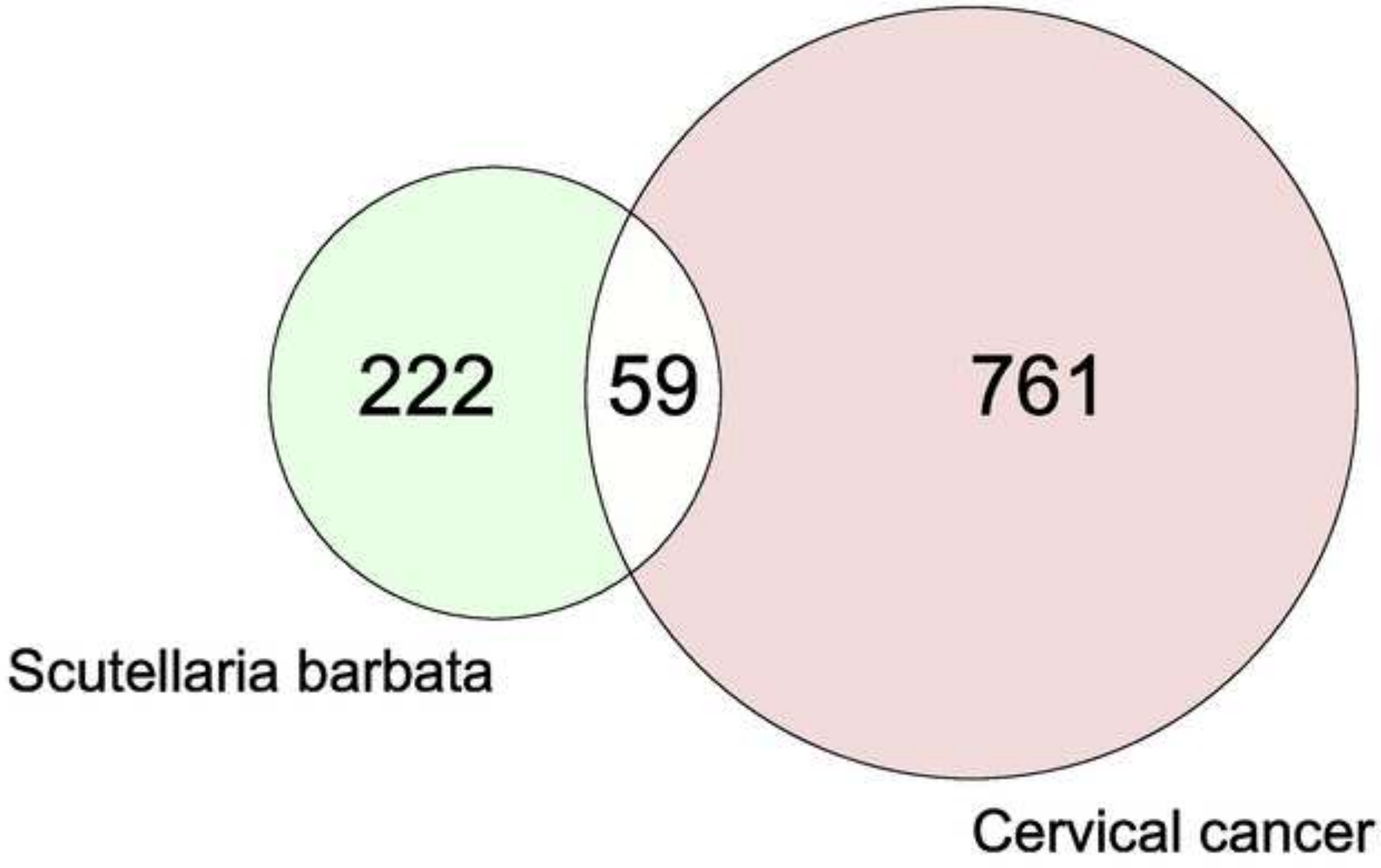

Figure 4

Click here to access/download;Figure;Figure 4.tiff

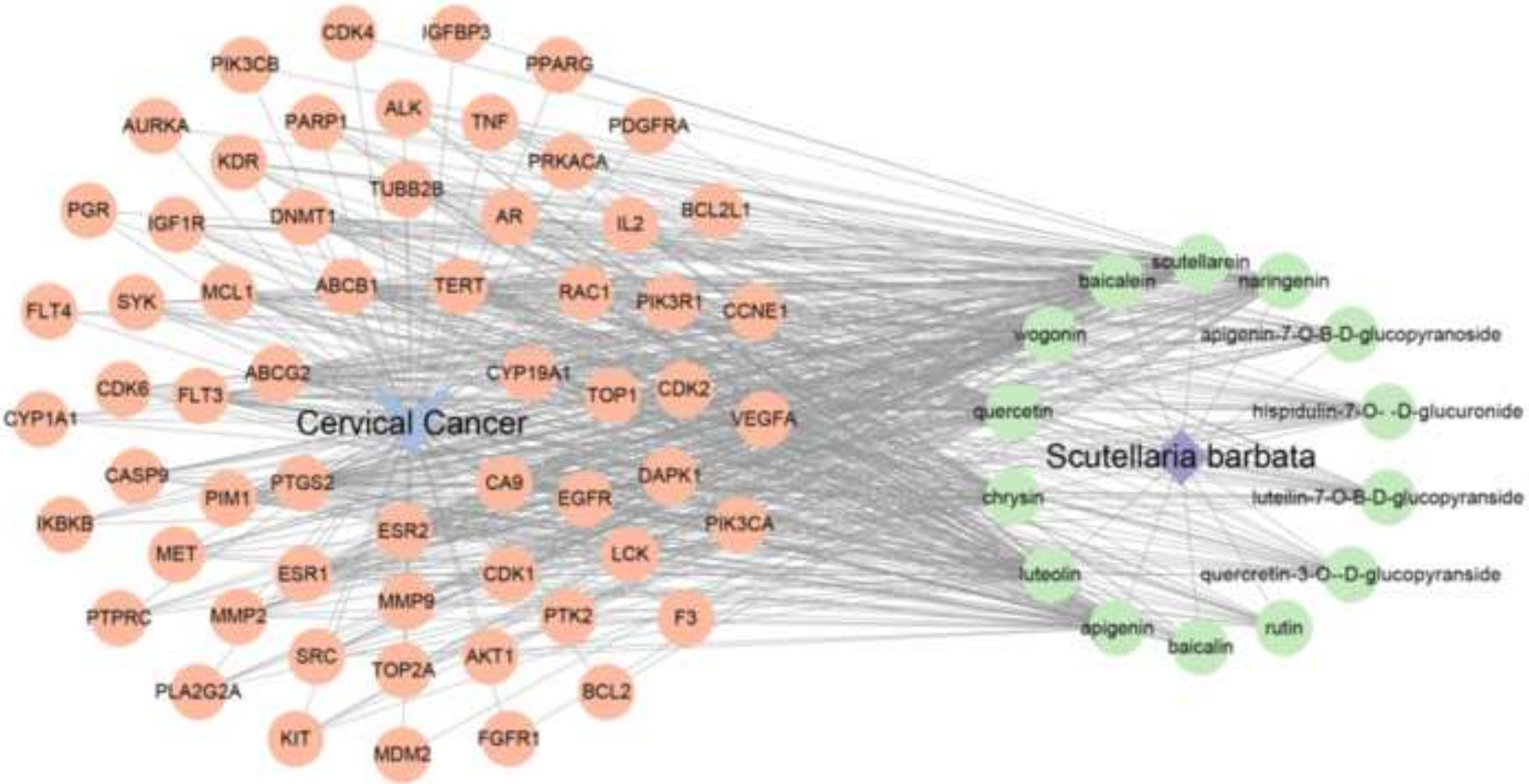

Figure 5

Click here to access/download;Figure;Figure 5 .tiff

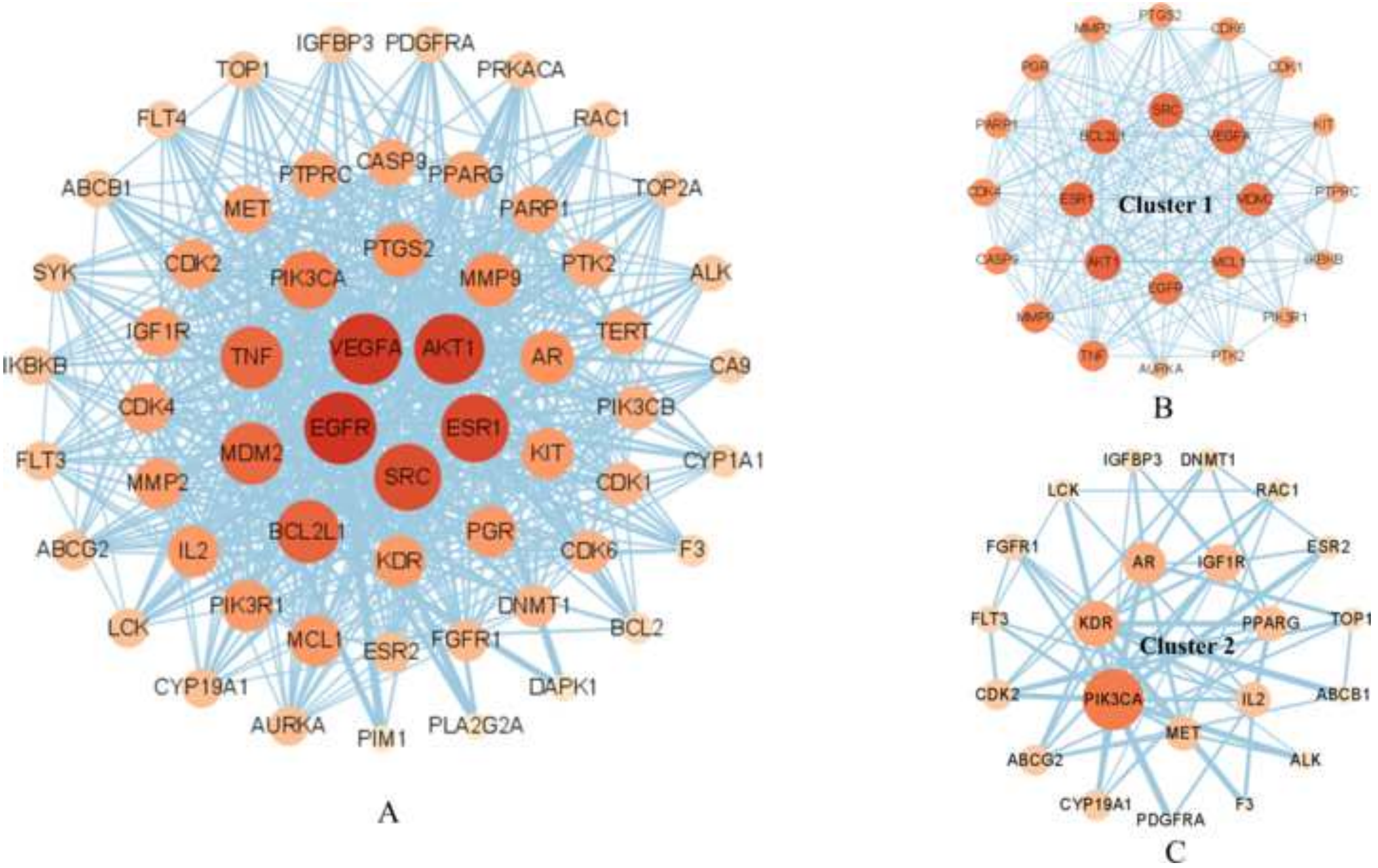

Figure 6

Click here to access/download;Figure;Figure 6.tif

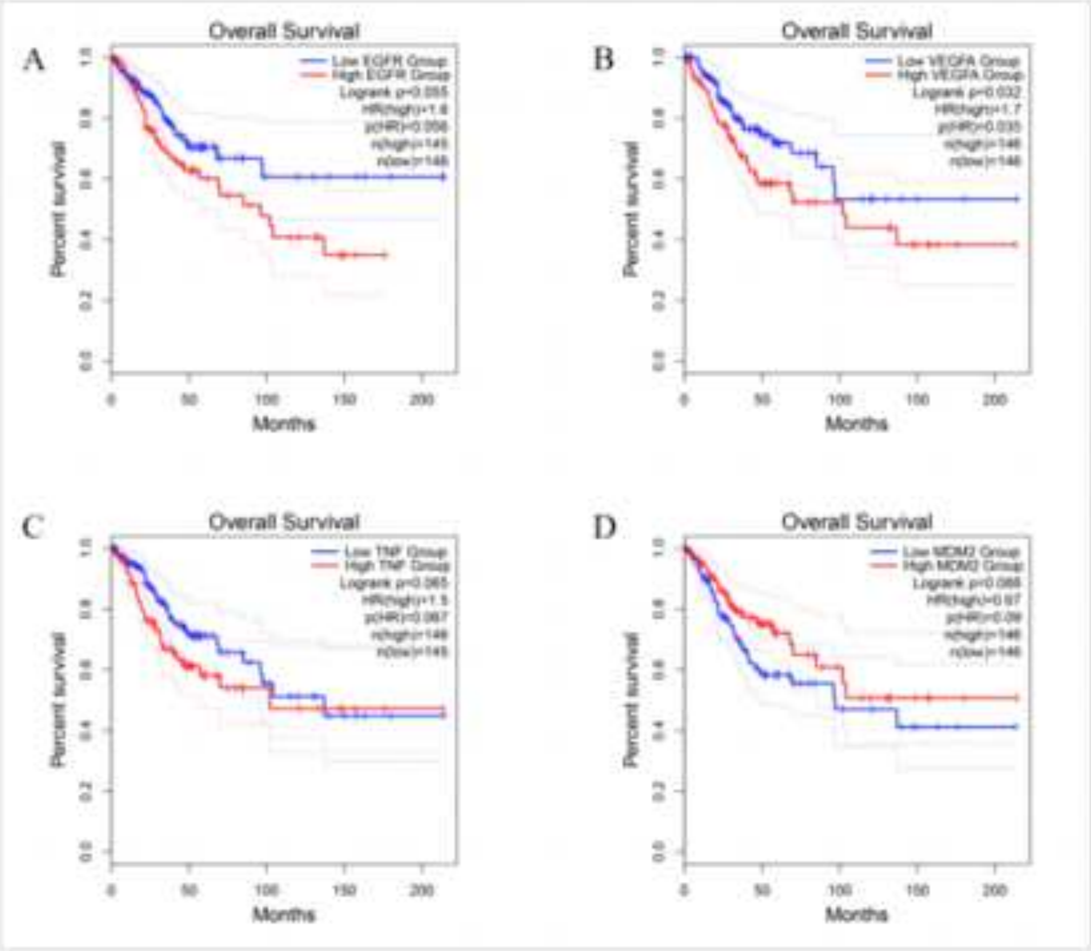

Figure 7

Click here to access/download;Figure;Figure 7.tiff

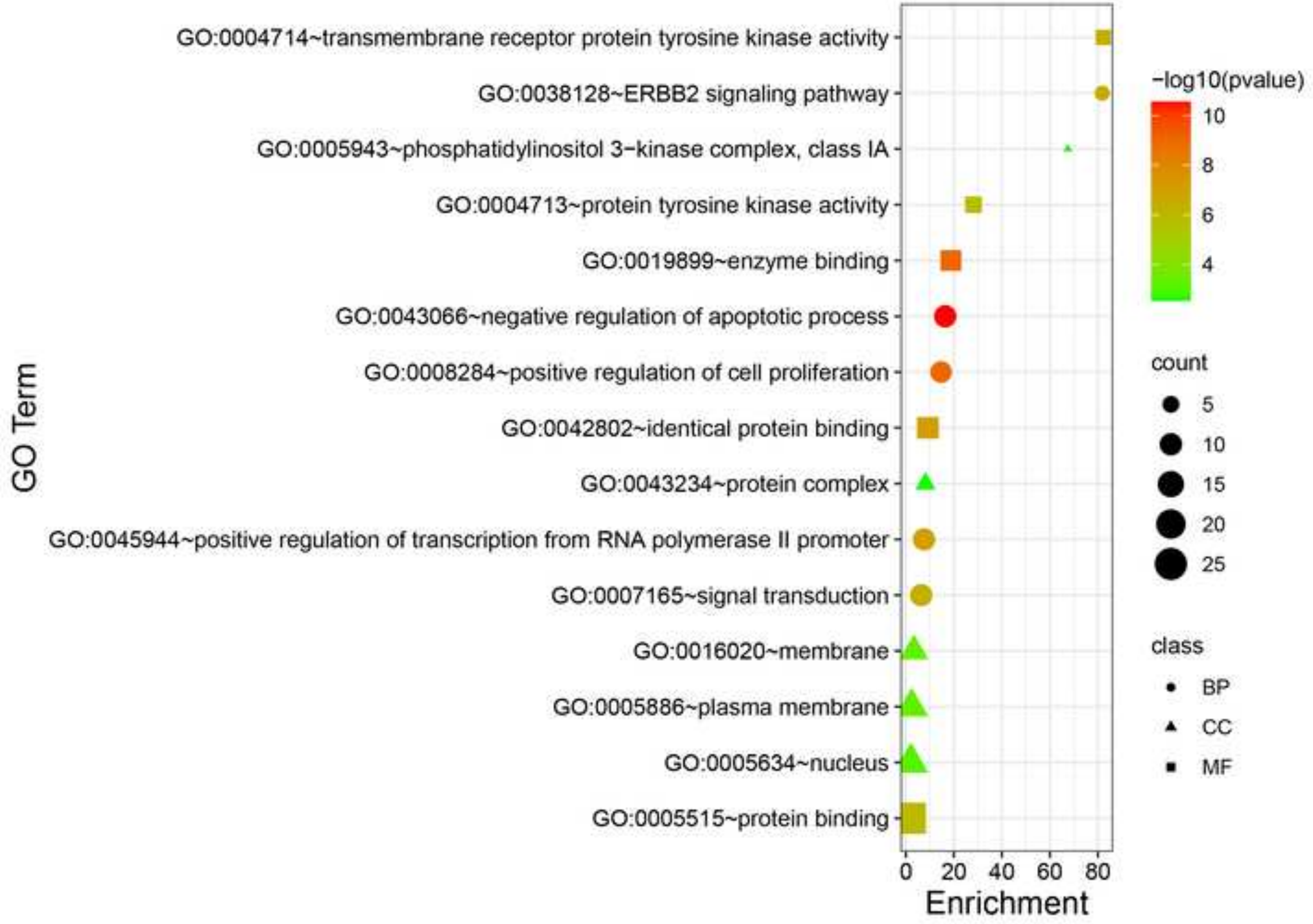

Figure 8

Click here to access/download;Figure;Figure 8.tiff

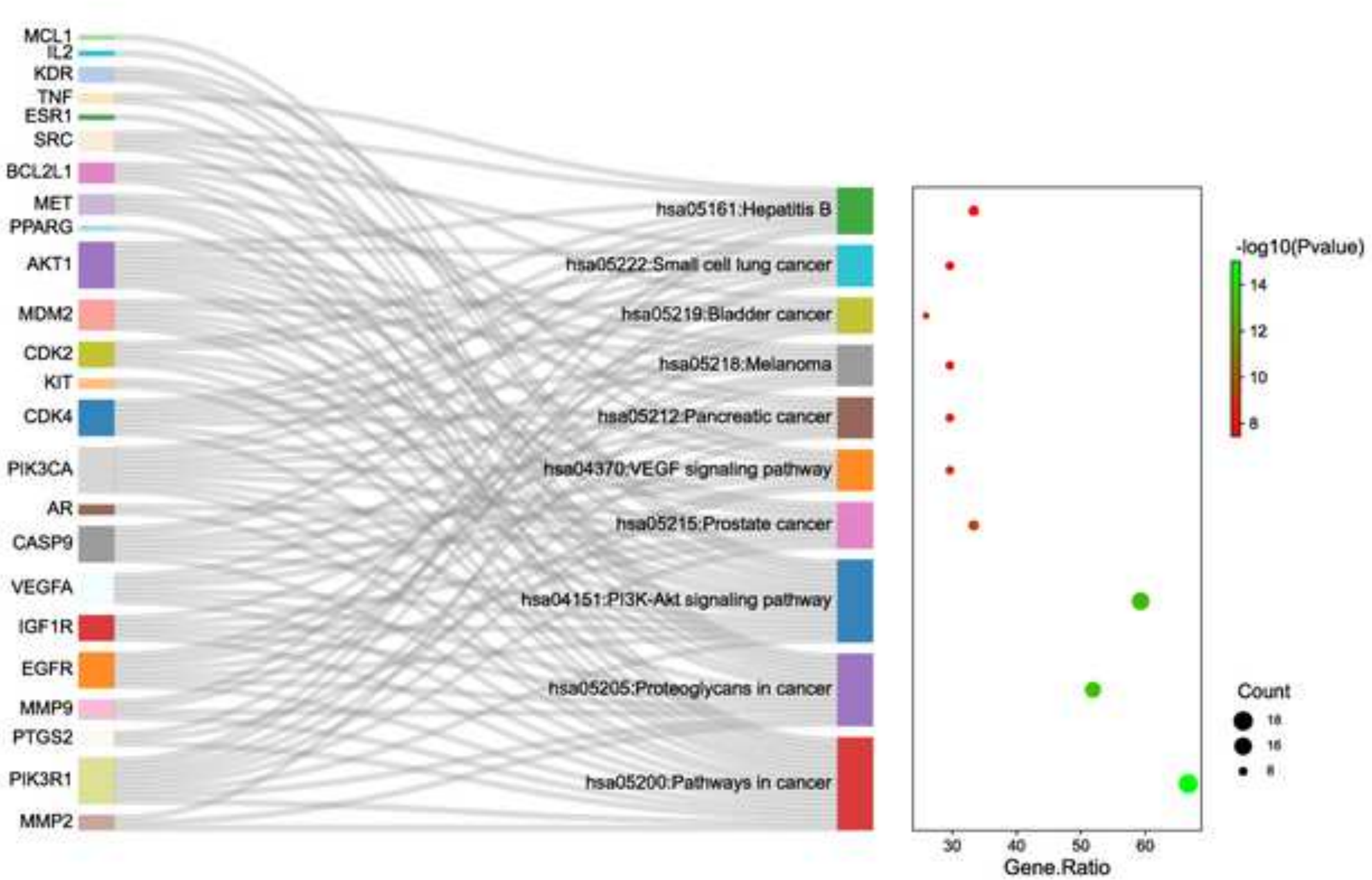

Figure 9

Click here to access/download;Figure;Figure 9.tiff

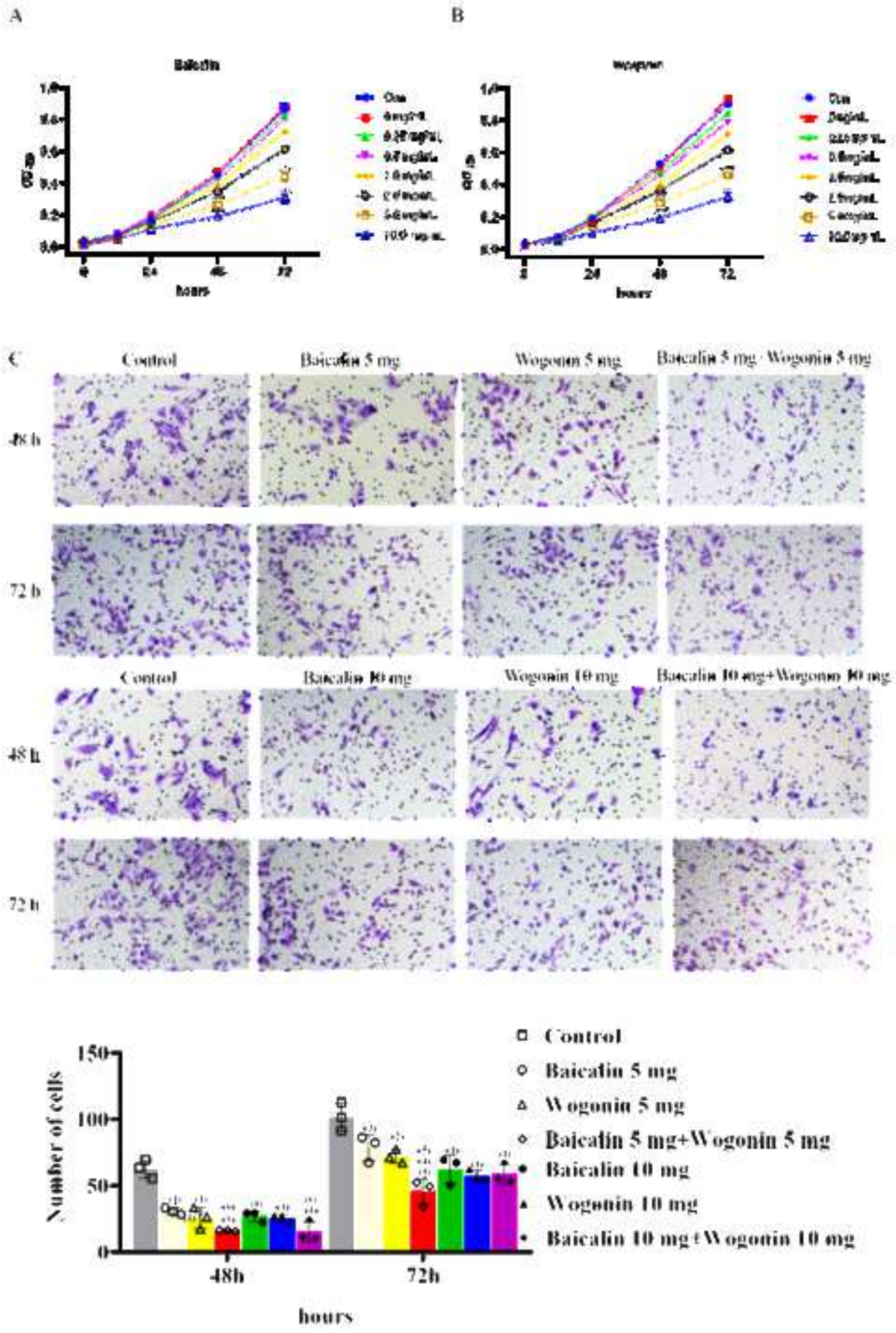

Figure 10 Click here to access/download;Figure;Figure 10.tiff

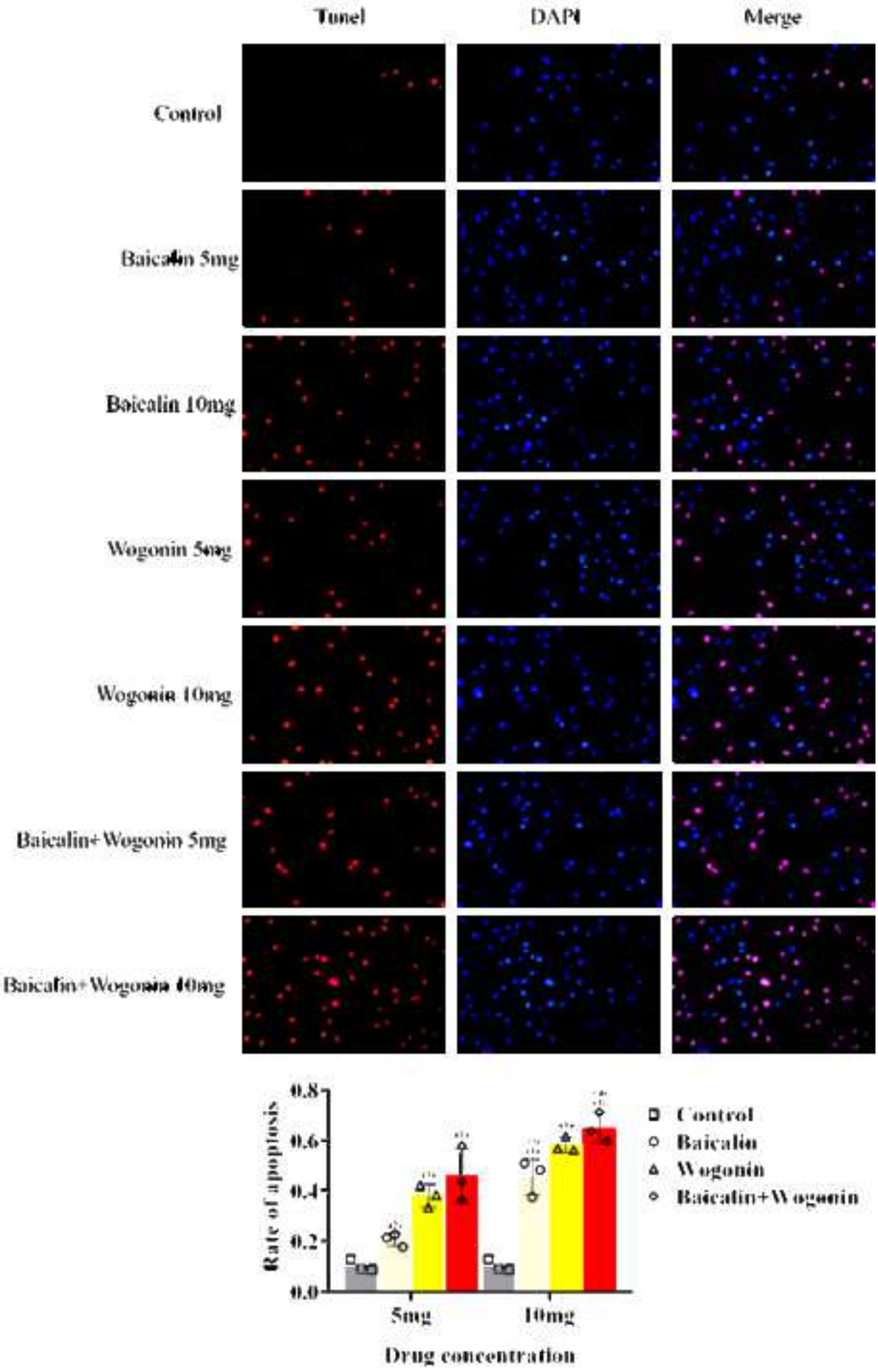

Figure 11

Click here to access/download;Figure;Figure 11.tiff

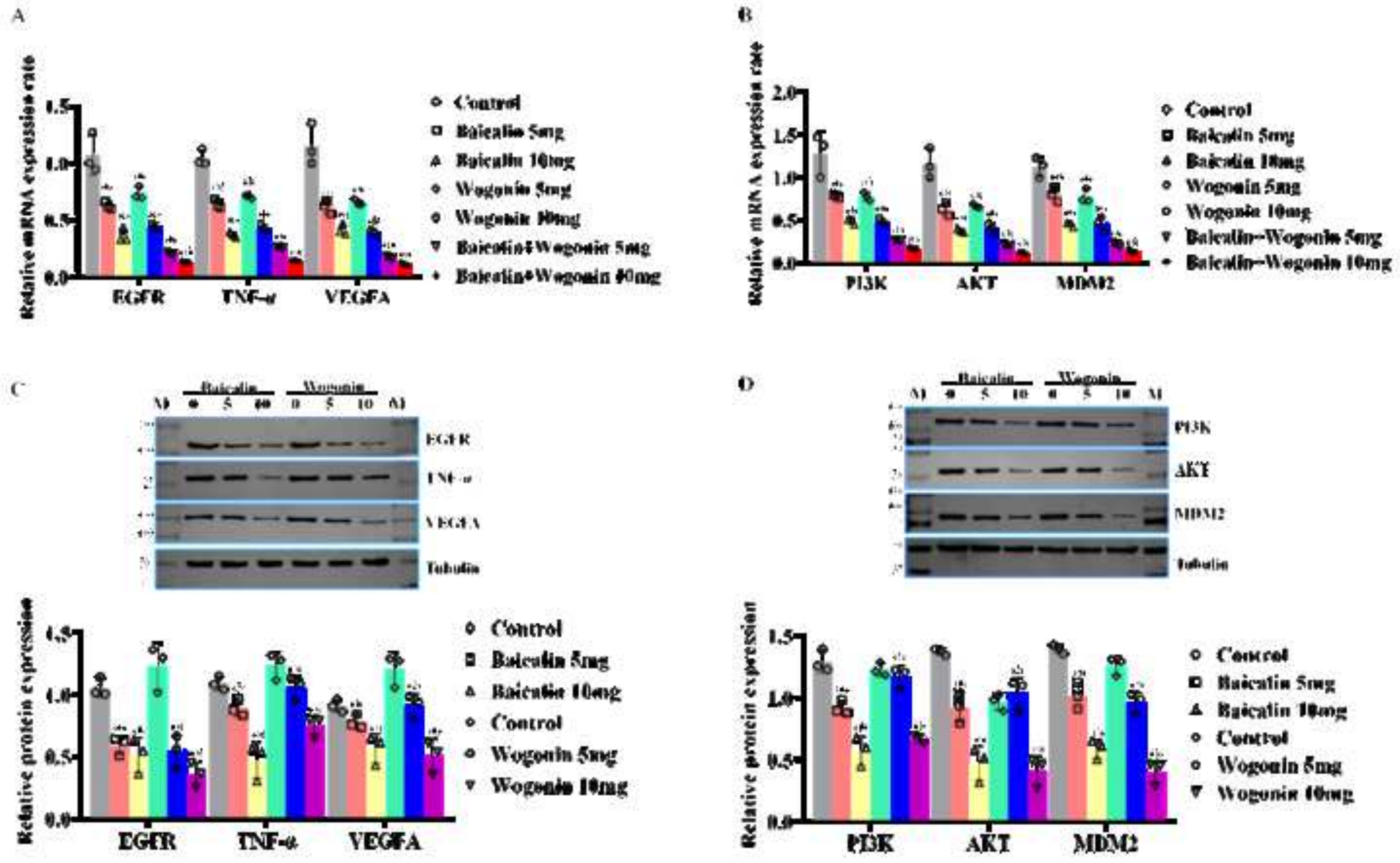

Click here to access/download;Figure;Figure12.tif

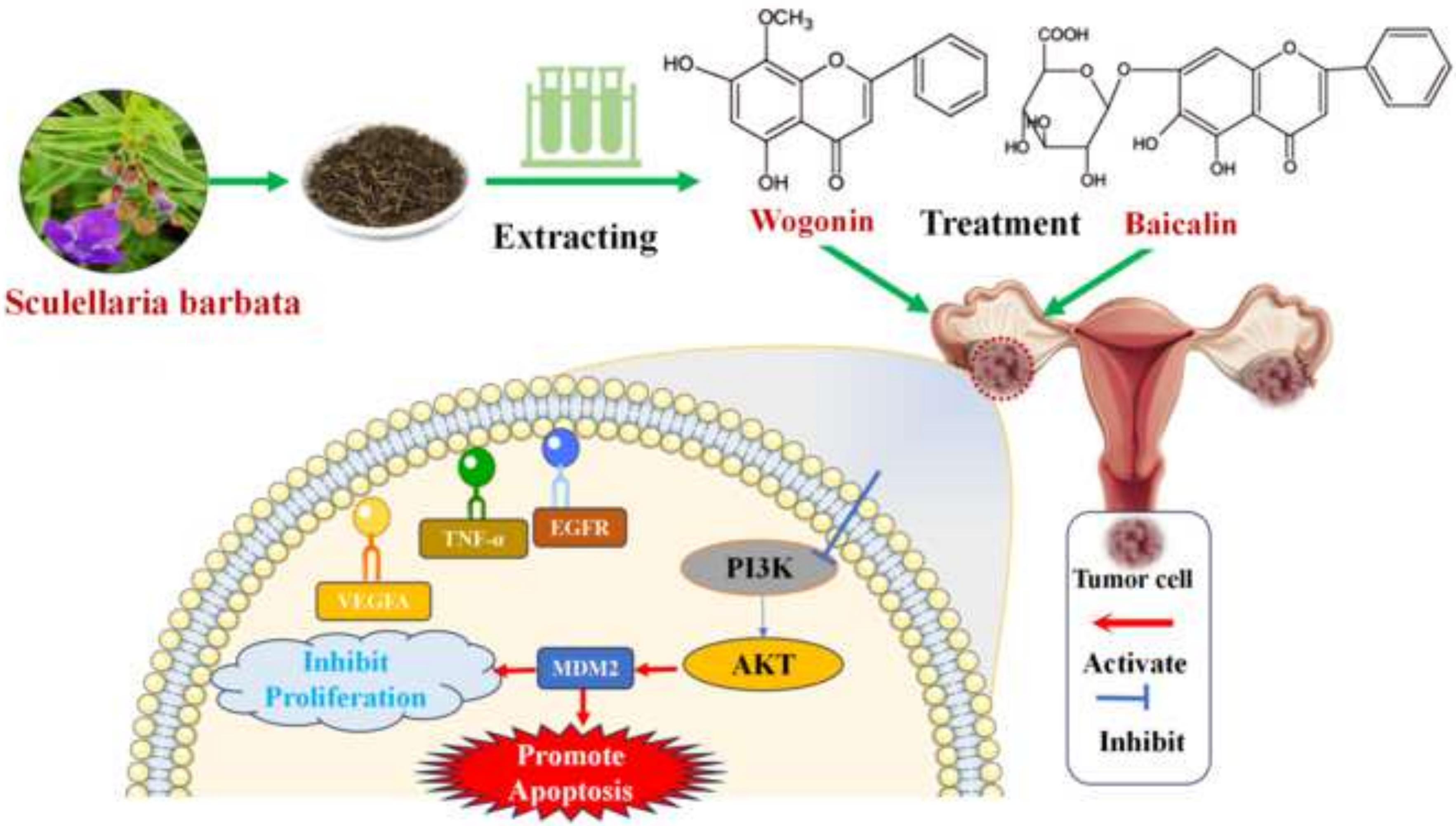

data sheet

Click here to access/download
Supporting Information
Data Sheet.docx
