# Supplementary material for: Scutellaria Barbata inhibits epithelial-mesenchymal transformation through PI3K/AKT and MDM2 thus inhibiting the proliferation, migration and promoting apoptosis of Cervical Cancer cells
Source: PLoS One. 2025 Apr 16;20(4):e0321556. doi: 10.1371/journal.pone.0321556 (PMC12002470; doi:10.1371/journal.pone.0321556)
Supplement: Attachment — Submitted filename: Review Report-PONE-D-24-33635.docx [file pone.0321556.s003.docx]

In the manuscript entitled “Scutellaria Barbata inhibits epithelial-mesenchymal transformation through

PI3K/AKT and MDM2 thus inhibiting the proliferation, migration and promoting

apoptosis of Cervical Cancer cells”, wen-qi feng et al have presented effect of Baicalein and Wogonin which are active compounds of Scutellaria Barbata. Overall, this work builds nicely on the previous reports about

the anti-tumor effects of Scutellaria Barbata, though there are some experimental issues that should be addressed, and some editing is required to better contextualize the meaning of these results. Discussion section should be improved. Authors are commended on assembling interesting scientific data; I look forward to reviewing a revised version of the manuscript soon.

Comments:

(1) Lines#33-37: Authors should re-write the sentences.
(2) If possible, date of access of websites for screening of active component should be mentioned.
(3) Method section 2.1.2-2.1.3 (Lines# 111-121): The English is very poor. It is better to improve whole sections.
(4) Line 134: Authors should correct **degree(degree)**.
(5) Method section 2.2: It is better to add method that how authors prepared and isolated component from the plant.
(6) Line#155: Please check and correct the sentence if required.
(7) Lines#165-166: Did the authors used the medium in liters?
(8) Line#204: It is difficult to understand. Author should change the heading so that readers can understand easily.
(9) Lines#212-213: It is difficult to understand. Authors should make a clear statement regarding common targets.
(10) Lines#237-238: Authors should re-write the outcomes of the experiments.
(11) Line#242: (after the addition of baicalin and **baicalein**,). Authors should correct it. Outcomes should be re-written.
(12) Line#246: There is confusion of active components. Authors should clarify baicalin and bacalealin. It is mentioned multiple times in the manuscript.
(13) Table 3: Table 3 should be divided into 3 parts. So that readers can understand easily. Or authors should prepare it again in a way that it is easy to understand.
(14) Line#284: This line should be checked and corrected.
(15) Conclusion section also needs to be improved.
(16) English language was ok but could be improved in some places. Large language models, such as chatGPT could be useful for the fluidity of the language, assuming that the authors meticulously check for the veracity of the statements.
